# Targeted detection of *Dehalococcoides mccartyi* microbial protein biomarkers as indicators of reductive dechlorination activity in contaminated groundwater

**DOI:** 10.1038/s41598-019-46901-6

**Published:** 2019-07-22

**Authors:** Manuel I. Villalobos Solis, Paul E. Abraham, Karuna Chourey, Cynthia M. Swift, Frank E. Löffler, Robert L. Hettich

**Affiliations:** 1 Chemical Sciences Division, Oak Ridge National Laboratory, Oak Ridge, Tennessee 37831 United States; 2 Department of Genome Science and Technology, University of Tennessee, Knoxville, Tennessee 37996 United States; 3 Department of Microbiology, Department of Civil and Environmental Engineering, Department of Biosystems Engineering and Soil Science, University of Tennessee, Knoxville, Tennessee 37996 United States; 4 Biosciences Division, Oak Ridge National Laboratory, Oak Ridge, Tennessee 37831 United States

**Keywords:** Environmental impact, Environmental sciences

## Abstract

*Dehalococcoides mccartyi* (*Dhc*) bacterial strains expressing active reductive dehalogenase (RDase) enzymes play key roles in the transformation and detoxification of chlorinated pollutants, including chlorinated ethenes. Site monitoring regimes traditionally rely on qPCR to assess the presence of *Dhc* biomarker genes; however, this technique alone cannot directly inform about dechlorination activity. To supplement gene-centric approaches and provide a more reliable proxy for dechlorination activity, we sought to demonstrate a targeted proteomics approach that can characterize *Dhc* mediated dechlorination in groundwater contaminated with chlorinated ethenes. Targeted peptide selection was conducted in axenic cultures of *Dhc* strains 195, FL2, and BAV1. These experiments yielded 37 peptides from housekeeping and structural proteins (*i.e*., GroEL, EF-TU, rpL7/L2 and the S-layer), as well as proteins involved in the reductive dechlorination activity (*i.e*., FdhA, TceA, and BvcA). The application of targeted proteomics to a defined bacterial consortium and contaminated groundwater samples resulted in the detection of FdhA peptides, which revealed active dechlorination with *Dhc* strain-level resolution, and the detection of RDases peptides indicating specific reductive dechlorination steps. The results presented here show that targeted proteomics can be applied to groundwater samples and provide protein level information about *Dhc* dechlorination activity.

## Introduction

*Dehalococcoides mccartyi* (*Dhc*) are key organohalide-respiring bacteria for the bioremediation of groundwater aquifers contaminated with industrial solvents such as tetrachloroethene (PCE) and trichloroethene (TCE). Chlorinated ethenes, including PCE and TCE, are common groundwater pollutants classified as toxic and carcinogenic to humans. Specialized *Dhc* bacteria grow under anoxic conditions by deriving energy from the reductive dechlorination of chlorinated ethenes, including *cis*-1,2-dichloroethene (*cis*-DCE) and vinyl chloride (VC), to ultimately yield environmentally benign ethene^1–5^. The ability of some *Dhc* strains to completely detoxify chlorinated ethenes makes them also functionally unique compared to other bacterial groups such as *Geobacter*, *Dehalobacter*, *Desulfitobacterium*, *Sulfurospirillum*, which comprise species that are not able to reduce PCE beyond *cis*-DCE^2^.

Various *Dhc* strains have been maintained in axenic cultures or in consortia supplied with a chlorinated ethene as electron acceptor, and several reductive dehalogenase (RDase) genes and their products have been identified as biomarkers of dechlorination activity^4,6,7^. Quantitative polymerase chain reaction (qPCR) measurements of the *Dhc* 16S rRNA gene and/or RDase genes in contaminated groundwater enabled comparative studies of the distribution and abundance of *Dhc* strains and RDase genes in response to bioremediation treatment (*i.e*., biostimulation and/or bioaugmentation)^8–10^. However, as with other existing nucleic acid-based measurement approaches, the challenge of qPCR measurements of 16S rRNA gene and/or specific RDase genes is their inability to reveal the actual metabolic activity. For example, several studies with mixed cultures have demonstrated the lack of significant correlation between dechlorination activity and the concentration of *Dhc* 16S rRNA genes^11,12^, temporal variation in the expression profiles of RDase transcripts during dechlorination^13,14^, as well as different degrees of RDase transcript correlation with dechlorination activity depending on substrate loading rates^13,15,16^.

Consequently, proteomics approaches to measure the expression levels of *Dhc* proteins involved in the reductive dechlorination process has been gaining traction. One of these approaches is targeted proteomics via liquid chromatography-multiple reaction monitoring-mass spectrometry (LC-MRM-MS), which enables the absolute quantification of proteins of interest by measuring proteotypic peptides derived from their enzymatic digestion^17^. LC-MRM-MS has been applied to pure and mixed cultures of *Dhc*,^7,18,19^; however, the utility of the approach for monitoring groundwater samples collected from sites impacted with chlorinated ethenes has not been demonstrated. Herein, we aimed to test the feasibility of developing and implementing a targeted proteomics approach via LC-MRM-MS for the detection of *Dhc* proteins to investigate *Dhc* reductive dechlorination activity in environmental groundwater samples from sites impacted with chlorinated ethenes.

To effectively track the presence of the targeted *Dhc* biomarker proteins, we first selected candidate proteotypic peptide sequences observed in high-mass-accuracy/high-resolution global proteomics datasets of actively dechlorinating pure cultures of *Dhc* strains 195, FL2, and BAV1. After signal evaluation of the selected peptides by LC-MRM-MS on a triple quadrupole mass spectrometer, the most robust and reproducible transitions (pairs of peptide precursor and fragment ions) were used to detect the targeted *Dhc* proteins in groundwater collected from six geographically distinct locations. Peptide identifications in groundwater samples were supported by comparing their fragmentation profiles to those obtained from pure cultures, or by comparing peak area differences, fragmentation profiles and retention times to spiked, unlabeled, synthetic peptide standards for further verification. Furthermore, 16S rRNA gene qPCR and global proteomics measurements performed for each groundwater sample allowed a comparative assessment with the LC-MRM-MS data.

## Results and Discussion

### Global proteomics measurements of axenic *Dhc* cultures inform peptide selection for targeted proteomics

**Figure 1:**
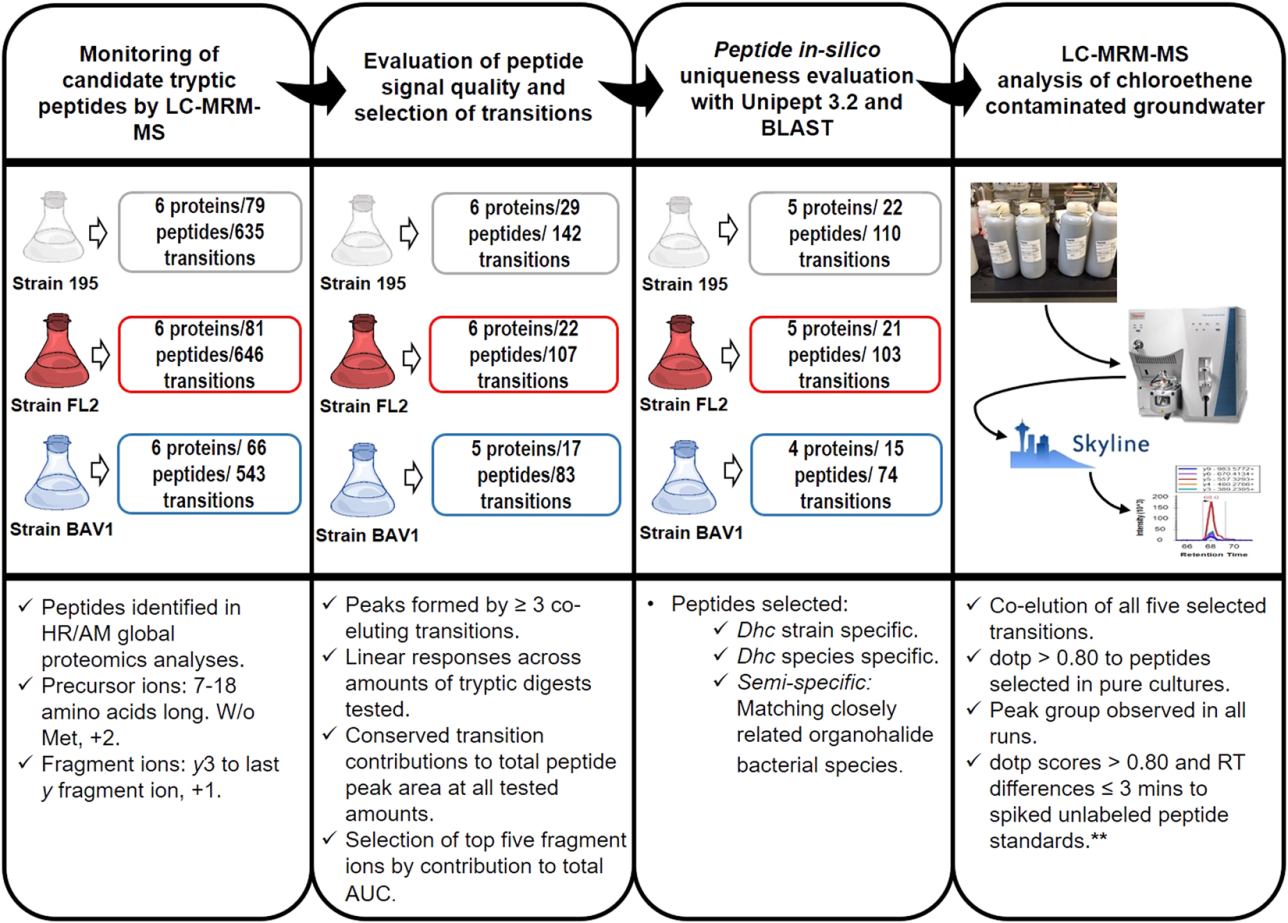
Workflow for the selection of peptide signals in pure cultures of *Dhc* strains 195, FL2 and BAV1. Each peptide peak group was submitted to a series of validation and refinement steps to identify peptide candidates having consistent fragmentation patterns, linearity in AUCs and ability to be generated upon tryptic digestion in groundwater monitoring. ** Only available for 11 targeted peptides.

The global proteomics datasets (Supplementary Table S1) collected from measurements of axenic *Dhc* cultures including *Dhc* strains 195, FL2, and BAV1 provided a set of candidate peptide sequences from housekeeping and reductive dechlorination biomarker proteins (Table 1) that were the starting point for the targeted method development (Fig. 1)^7,18,19^.

**Table 1:** *Dhc* protein biomarkers used as initial targets in this study.

Targeted biomarker [Designation]	Biomarker description	Strain 195^a^	Strain FL2^b^	Strain BAV1^a^
60 kDa chaperonin [GroEL]	Housekeeping protein. Informs presence of *Dhc*.	Q3Z6L3	demc_1274	ABQ17815
Formate dehydrogenase, alpha subunit [FdhA]^c^	General marker of active dechlorination processes.	Q3ZA14	demc_808	ABQ16756
Trichloroethene reductive dehalogenase [TceA]	Process specific marker of active dechlorination (TCE→VC)	Q3ZAB8	demc_738	×^d^
Vinyl chloride reductive dehalogenase [BvcA]	Process specific marker of active dechlorination (DCEs, VC → Ethene)	×	×	ABQ17429
Elongation factor Tu [EF-TU]	Housekeeping protein. General activity/presence of *Dhc*.	Q3Z7S9	demc_108	ABQ17463
Ribosomal protein L7/L12 [rpL7/L12]	Housekeeping protein. General activity/presence of *Dhc*.	Q3Z7T6	demc_114	ABQ17470
BNR/Asp-box repeat domain protein [S-layer]	Structural protein. Presence of *Dhc*.	Q3Z6N3	demc_1296	ABQ17793

^a^Protein databases from *Dhc* strains 195 and BAV1 were downloaded from Uniprot (IDs. UP000008289 and UP000002607, respectively). ^b^The IGS Annotation Engine was used for structural and functional annotation of the *Dhc* strain FL2 sequences (http://ae.igs.umaryland.edu/cgi/index.cgi, Reference: PMID:21677861) and the web-based tool Manatee was used to view and download protein annotations (http://manatee.sourceforge.net/). ^c^*Dhc* bacteria are unable to grow using formate. Cells extracts lack any formate dehydrogenase ability. Recent work has assigned the FDH protein and its subunits an electron transfer role to the RDases during reductive dechlorination reactions. See Kublik *et al*.^30^ for more details. ^d^× - protein is not present in the respective proteome.

**Figure 2:**
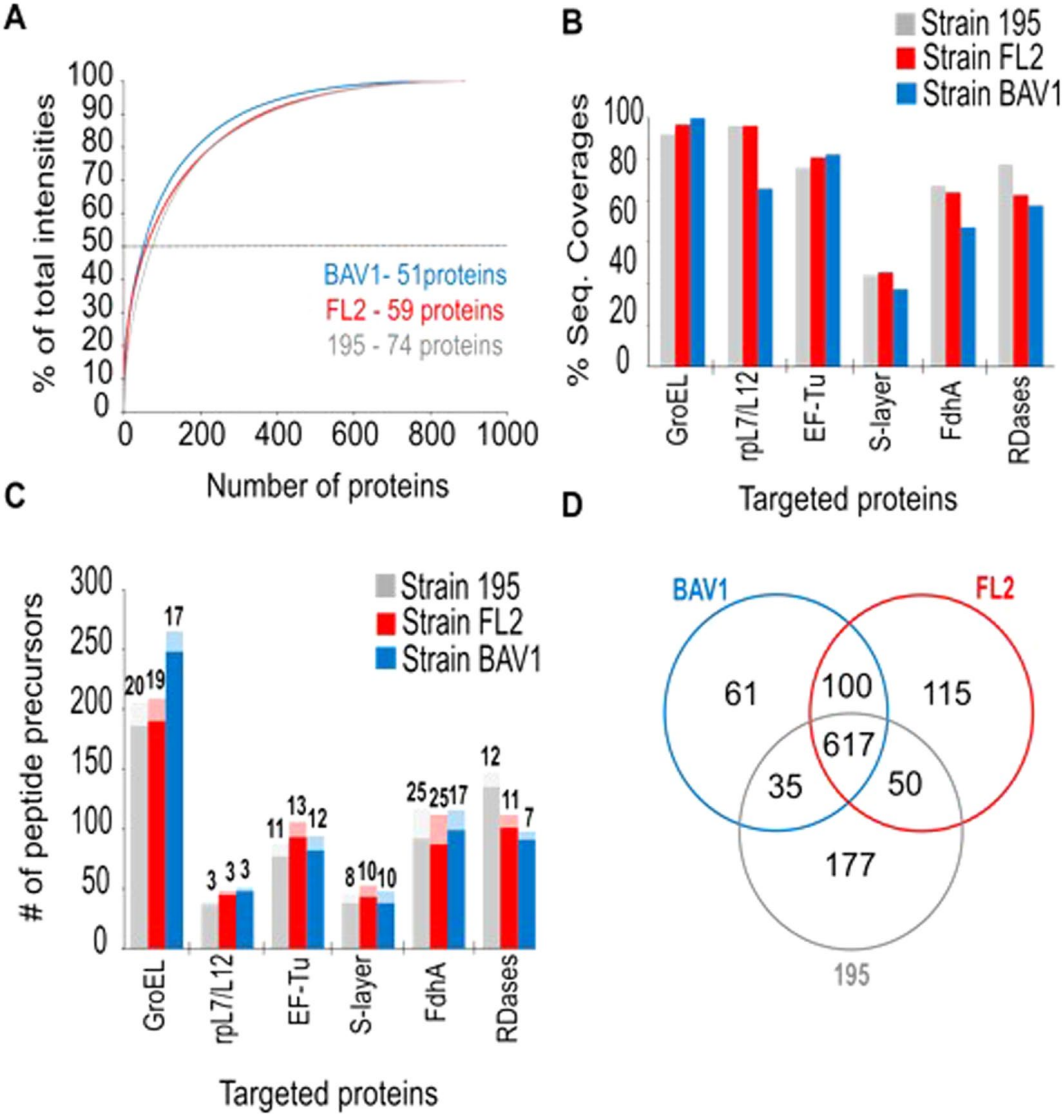
Global proteomics metrics from the analyses of three pure cultures of *Dhc*. (**A**) Relative percentage of contribution to the total intensity of the proteins identified with a peptide-level confidence >99% in the global proteomic analyses of axenic cultures of *Dehalococcoides mccartyi* strains 195, FL2 and BAV1. Targeted proteins ranked amongst the numbers of proteins contributing to half of the total measured intensities (below the dashed line, numbers next to strain names). (**B**) Targeted protein sequence coverages obtained from the cultures processed in this study. (**C**) Total number of peptide spectrum matches (PSMs) for each targeted protein. RDases per strain are homologues of TceA in strain 195 and FL2, and BvcA in strain BAV1. The numbers in the bar chart (represented by light colors) are the fraction of +2 peptide charged precursors meeting the selection criteria for LC-MRM-MS analysis described in Materials & Methods. (**D**) Number of protein groups (>85% sequence identity) identified in each culture.

Global proteomics analyses resulted in proteome coverages of 59%, 57%, and 60% for *Dhc* strains 195, FL2, and BAV1, respectively. These percentages are close to the ~60% that has been obtained before in shotgun proteomics studies of *Dhc* strains 195, CBDB1, and DCMB4^18,20–22^. Overall, the analytical dynamic range of the proteome measurements spanned ~5 orders of magnitude in terms of protein intensities (based on summed peptide peak areas). All the targeted housekeeping and structural proteins (*i.e*., chaperonin GroEL, S-layer associated protein) and those indicative of active dechlorination (*i.e*., FdhA, TceA, and BvcA) ranked amongst the top 50% most abundant proteins (Fig. 2A). Each biomarker was also found with similar normalized intensities within each strain dataset (Supplementary Fig. S1). The resulting percentages of sequence coverages and the number of peptide precursors per targeted protein demonstrated comparable efficiencies of tryptic digestion achieved between the *Dhc* strains included in the analysis (Fig. 2B,C).

The expression of FdhA proteins observed in cultures of strains 195, FL2, and BAV1 is in agreement with prior studies that have reported observed FdhA in comparable abundances relative to RDases and hydrogenases in actively dechlorinating *Dhc* pure and mixed cultures^23–27^. In addition, measured mRNA levels of the Fdh subunits have been reported to be dependent on the presence of a chlorinated electron acceptor but not on the presence of the electron donor hydrogen^28^. Recent studies of the Fdh complex (i.e., the iron-sulfur molybdoenzyme complex I [CISM]) of *Dhc* strain CBDB1 revealed a tight spatial association between FdhA and the RDase CbrA (ID. CbdbA194)^29^. Supported by *in-vitro* dehalogenation activity assays, these observations suggest that FdhA serves an integral role in the respiratory chain of *Dhc* (*i.e*., FdhA may serve as an electron-channeling module between the Hup hydrogenase and the RDase)^30^, and as such, can serve as a general biomarker of *Dhc* dechlorination activity.

RDase enzymes are biomarkers of active dechlorination and can provide additional information regarding specific chlorinated compounds that undergo reductive dechlorination. The types of chlorinated compounds dechlorinated by various RDases makes the *Dhc* group functionally diverse^24,31–33^. The TceA RDase in the proteomes of strains 195 and FL2 and the BvcA RDase in the proteome of strain BAV1 were observed amongst the top five most abundant proteins in their global proteomics dataset, respectively (Supplementary Table S1).

Sequence identities of the protein biomarkers selected for each strain were also evaluated. In total, 617 protein groups (>85% amino acid sequence identity) were common between the three *Dhc* strains analyzed (Fig. 2D). These protein groups encompass homologues of the targeted GroEL, EF-TU, rpL7/L12, and FdhA proteins. Interestingly, the putative S-layer sequence of strain BAV1 and the annotated S-layer proteins of strains 195 and FL2 did not group together. The TceA homologues of strains 195 and FL2 clustered at 99% identity, while RDase BvcA was found amongst the 61 unique protein groups of strain BAV1. These observations reveal that candidate peptide sequences from protein biomarkers can target multiple *Dhc* strains or can be potentially used as strain-specific targets when monitoring mixed cultures or environmental samples.

In addition to the TceA homologues and BvcA, 18 other RDases were identified in these shotgun proteomics measurements, albeit at lower abundances (Supplementary Fig. S2). The protein sequence coverages of the other identified RDases were on average below 60%, except for two other RDases (demc_816 in strain FL2 and Q3Z6A6 in strain 195). The identification of multiple RDases in actively dechlorinating *Dhc* cultures is related to the various sets of RDase genes present in single *Dhc* genomes (*i.e*., 17 RDase genes in strain 195, 24 RDase genes in strain FL2, and 11 RDase genes in strain BAV1^27^). The co-expression of RDases by single *Dhc* strains has been reported and has been hypothesized as a mechanism of adaptation to use naturally occurring and anthropogenic organohalogens^34,35^.

To provide insight into the diversity of the expressed RDases and validate the biomarker selection, we evaluated the phylogenetic relationships of the RDases present in the proteomes of *Dhc* strains 195, FL2, and BAV1. The selected TceA homologues from strains 195 and FL2 formed a sub cluster, while the targeted BvcA did not group with any of the RDases, nor did any of the second most abundant RDases in each dataset cluster with any of the targeted enzymes (Supplementary Fig. S2). The phylogenetic analysis demonstrated the sequence conservation of TceA and BvcA as compared to other RDases expressed by other or the same *Dhc* strains. Moreover, the substrate ranges of TceA homologues and BvcA are known, while the participation of other RDases in reductive dechlorination reactions remains to be determined experimentally^27^. The higher expression and sequence coverages obtained for the TceA and BvcA RDases resulted on average in four times higher numbers of tryptic peptides than those obtained for other expressed RDases, which was helpful for the development of the targeted assay.

### Selection of *Dhc* MRM-MS observable peptides and *in-silico* evaluation of their biological specificities

Evaluation by LC-MRM-MS was conducted on 79, 81, and 66 peptides from the seven targeted proteins of *Dhc* strains 195, FL2, and BAV1, respectively (Table 1) that had been identified in the global proteomics datasets. By examining three different loading amounts of total digested protein (i.e., 500 ng, 2 µg, and 8 µg) and manually analyzing the data to determine the quality of the resulting peptide signals, 29 peptides and 142 transitions from the digest of strain 195, 22 peptides and 107 transitions from strain FL2, and 17 peptides and 83 transitions from strain BAV1, were selected (Fig. 1). Examples of the type of signals chosen and discarded from these steps are shown in Supplementary Figs S3 and S4. From this initial selection, the top five transitions ranked by contribution to total area under the curve (AUC) per peptide were preserved, resulting in a total of 55 peptides (unique and shared between strains) equivalent to 270 transitions.

Essential for targeted proteomics experiments are control measures to ensure that the selected peptides uniquely identify the protein(s) of interest^17^. The microbial diversity, and hence the diversity of proteins, in particular the presence of other organohalide-respiring bacteria thriving in the environments where *Dhc* is found^2,36,37^, creates a challenge for the selection of unique peptides. An *in-silico* comparison between several *Dhc* proteomes, as well as the proteomes of other organohalide-respiring bacteria commonly found in groundwater aquifers or sediments, demonstrated that *Dhc* strains shared greater similarities (≥47%) amongst their peptidomes compared with those of other bacterial species (≤4% similarity between the peptidomes of *Dhc* strains 195 and VS with *Dehalogenimonas lykantroporepellens* strain BL-DC-9) (Supplementary Fig. S5). Although this analysis supported the development of a species-level targeted proteomic assay for *Dhc* and its application to contaminated groundwater, the sequence specificities of each selected peptide candidate were further assessed individually with the Tryptic Peptide Analysis tool of Unipept 3.2 and Protein BLAST searches. Peptides were deemed as *Dhc*-specific when they were not found in any other bacterial protein sequence available in UniProt and NCBI *nr* databases, and when multiple *Dhc* strains shared the candidate peptide sequence by means of both*-silico* searches. Peptides were considered semi-specific when they were found in proteins derived from related organohalide-respiring bacteria, and as non-specific when they were found in proteins of non-organohalide respiring bacteria. Compiled results from these *in-silico* searches are presented as Supplementary Table S2.

Out of the seven peptides selected for monitoring the presence of the housekeeping chaperonin GroEL, peptide DGVITIEESR was the only one non-specific to *Dhc*. Considering that homologues of this housekeeping protein are found in diverse bacteria^38^, it was surprising that six *Dhc*-unique peptides could be identified. From the targeted EF-TU proteins, peptide TTLTAAITR was found in more than 100 UniProt protein entries, and similar observations were made for peptide ELTSLGLK from the ribosomal protein L7/L12. The presence of peptides DGVITIEESR, TTLTAAITR, and ELTSLGLK in the proteomes of non*-*organohalide respiring bacteria prompted us to remove them from the list of selected peptides, which resulted in the loss of the rpL7/L12 marker protein.

Candidate peptides from the annotated FdhA (general biomarker of *Dhc* activity) and S-layer (structural housekeeping) proteins were specific to *Dhc* and in certain cases provided strain level resolution. For example, the *in-silico* analysis demonstrated that the FdhA peptides GTELISVDCR and SELEVISSLFSR were specific to *Dhc* strain 195, while peptide TDNNTNYSYINAIK was specific to the FdhA of *Dhc* strain BAV1. All peptides of the S-layer protein were specific to a few *Dhc* proteomes stored in UniProt, a useful characteristic for environmental monitoring of certain *Dhc* strains.

The expression of RDases from bacteria other than *Dhc* can complicate their *exclusive* use as specific biomarkers of *Dhc*-mediated reductive dechlorination in groundwater^16^. For example, the *in-silico* searches of the six peptides selected in total for TceA and BvcA RDases revealed that these are also found in RDase sequences of other organohalide-respiring bacteria like *Dehalogenimonas*. Thus, the information that the shared RDase peptides selected here could provide in contaminated groundwater needs to be interpreted in concert with information from other biomarker proteins, such as FdhA, to have a more direct line of evidence that *Dhc*-specific biologically driven dechlorination is occurring at a site. Additionally, the identification of shared RDase peptides in an MRM assay, combined with other experimental measurements like *Dhc* 16S rRNA gene-targeted qPCR, can provide insights into the identity of the bacterial species carrying out dechlorination processes. Altogether, these observations suggested that a panel of protein biomarkers should be utilized for the most detailed characterization of *Dhc* mediated dechlorination processes in groundwater. Supplementary Table S3 shows the complete list of peptides and their transition *m/z* values per protein used for LC-MRM-MS analysis of groundwater.

### Application of the selected biomarkers for targeted proteomics analyses in a PCE-to-ethene-dechlorinating consortium

Peptides and transitions selected in axenic *Dhc* cultures were initially tested in a tryptic digest of the nonmethanogenic PCE-to-ethene dechlorinating BDI consortium. BDI harbors multiple *Dhc* strains, including strains BAV1, FL2, and GT^3,39^. The known microbial diversity of BDI allowed an easier validation of peptide identification with criteria that included, amongst others, the comparison of dot-product (dotp) correlation scores for transition intensity ratios between the signals detected in samples to those observed in pure cultures or to samples spiked with 5 pmol of internal standards (Supplementary Table S4).

**Figure 3:**
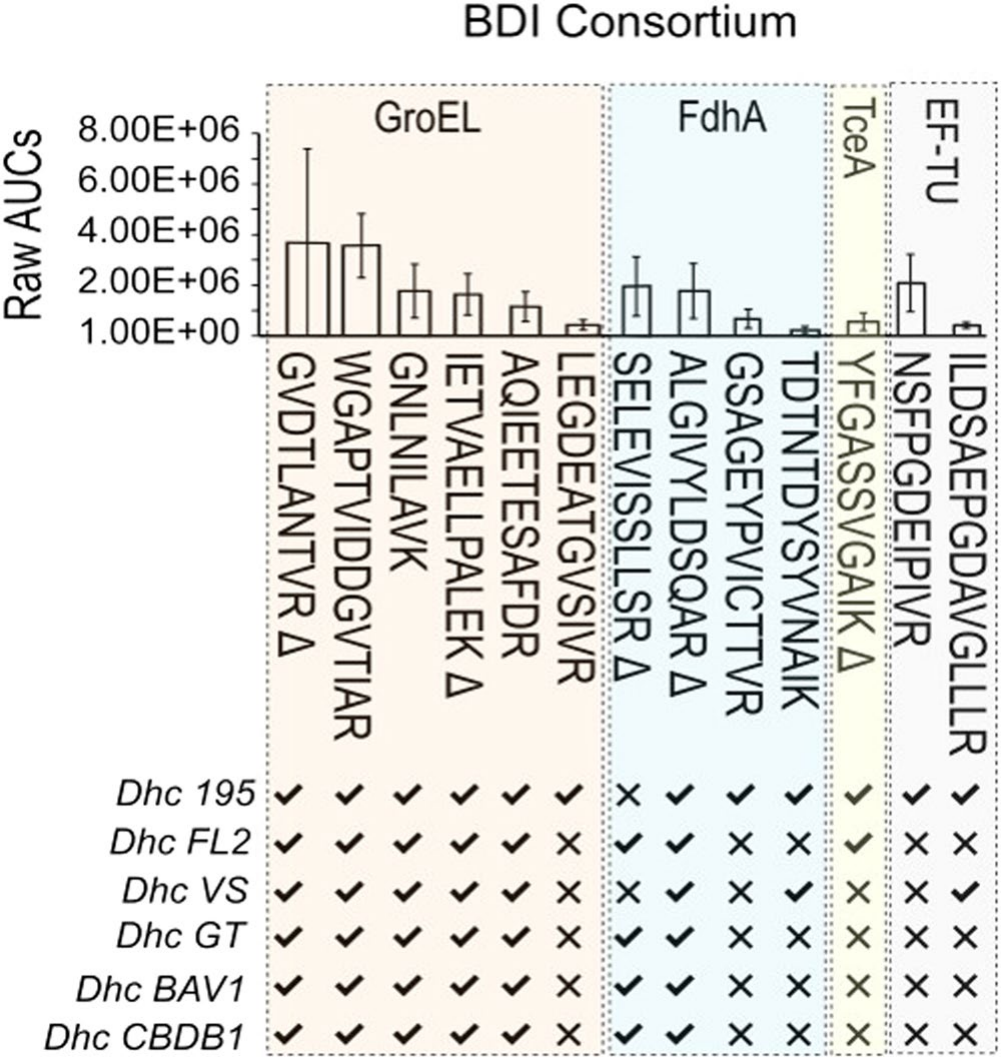
LC-MRM-MS *Dhc* biomarker identification in a tryptic digest of the PCE-to-ethene dechlorinating consortium BDI. The Fig. shows the average raw peak area under the curve (AUC) values of the targeted peptides identified in three technical replicate LC-MRM-MS runs. Error bars are the standard error of the mean. Peptides marked with Δ were identified with supporting evidence from spiked-in unlabeled standards. The inserts below the graph show the specificities of the peptides, determined *in-silico*, to the proteomes of the six most common isolates of *Dhc* bacteria. The complete list of other proteins and organisms that can produce the same peptide upon tryptic digestion are listed in the Supplementary Table S2.

Through LC-MRM-MS analyses, 13 peptides were identified out of the 37 that were targeted. Among these, GroEL peptides with high representation in the proteomes of multiple strains of *Dhc* were observed (Fig. 3). However, we also detected the more conserved GroEL peptide LEGDEATGVSIVR, which, according to the UniPept searches, is only present in the proteomes of *Dhc* strains 195, KBTCE2, CG4 and KBTCE3 (Supplementary Table S2). Interestingly, in relation to the identification of peptide LEGDEATGVSIVR, we also detected the EF-TU peptide NSFPGDEIPIVR, which is specific to the proteomes of the same *Dhc* strains as peptide LEGDEATGVSIVR, thus suggesting that these strains are part of the *Dhc* population in consortium BDI.

Active dechlorination activity was inferred through the presence of four FdhA peptides and one of the targeted TceA peptides. As with the identification of the GroEL peptide LEGDEATGVSIVR and the EF-TU peptide NSFPGDEIPIVR, the FdhA peptide GSAGEYPVICTTVR also found in the proteomes of strains 195, KBTCE2, CG4 and KBTCE3 suggested the involvement of one or more of these strains in the dechlorination process. In addition, the detection of the TceA peptide YFGASSVGAIK, shared by *Dhc* strains 195 and FL2, provided additional evidence for the presence of strain 195 in culture BDI.

The evidence provided by targeted proteomics about the existence of additional but not yet recognized *Dhc* strains in consortium BDI prompted us to explore the microbial diversity of this culture by means of high-mass-accuracy/high- mass-resolution global proteomics analyses. By assembling a proteome database of other known strains of *Dhc* (Supplementary Table S5), the BDI spectral data indeed revealed that organisms representing *Dhc* strain 195 were present this culture, as we were able to detect unique peptides matching proteins specific to certain strains (*i.e*., to the S-layer protein of *Dhc* strain 195). The complete list of protein identifications in BDI is presented in Supplementary Table S7.

Global proteomics analyses also revealed the absence of the BvcA enzyme in BDI, which agreed with the targeted results. This information was also corroborated with prior qPCR experiments showing that *Dhc* strain BAV1 carrying the *bvcA* gene was lost from consortium BDI after repeated transfers with PCE or TCE^2^. The lack of *Dhc* bacteria expressing BvcA in BDI seems to be compensated by *Dhc* strains expressing VcrA (*i.e*., strains GT and VS). VcrA was not targeted in the MRM assay, but expression levels of this enzyme were confirmed by global proteomics in consortium BDI, where it may play a role in the dechlorination of *cis*-DCE to ethene. The involvement of microorganisms expressing VcrA, was also supported by the targeted detection of FdhA peptides matching to the proteomes of *Dhc* strains VS and GT (Fig. 3).

### Application of the selected biomarker panel for targeted proteomics analyses of groundwater impacted with chlorinated ethenes

Seven groundwater samples collected from various international sites impacted with chlorinated ethenes were analyzed by targeted proteomics. Amongst the identified contaminants were TCE, *cis*-DCE and VC. These compounds are substrates and intermediates of the anaerobic reductive dechlorination reactions carried out by *Dhc* bacteria that ultimately yield ethene as the end product. Ethene was detected in these samples at various concentrations. The detection and concentrations of these chemicals provide some level of information about the degree of dechlorination, in each sample, and are tabulated for each groundwater sample in Supplementary Table S6).

**Figure 4:**
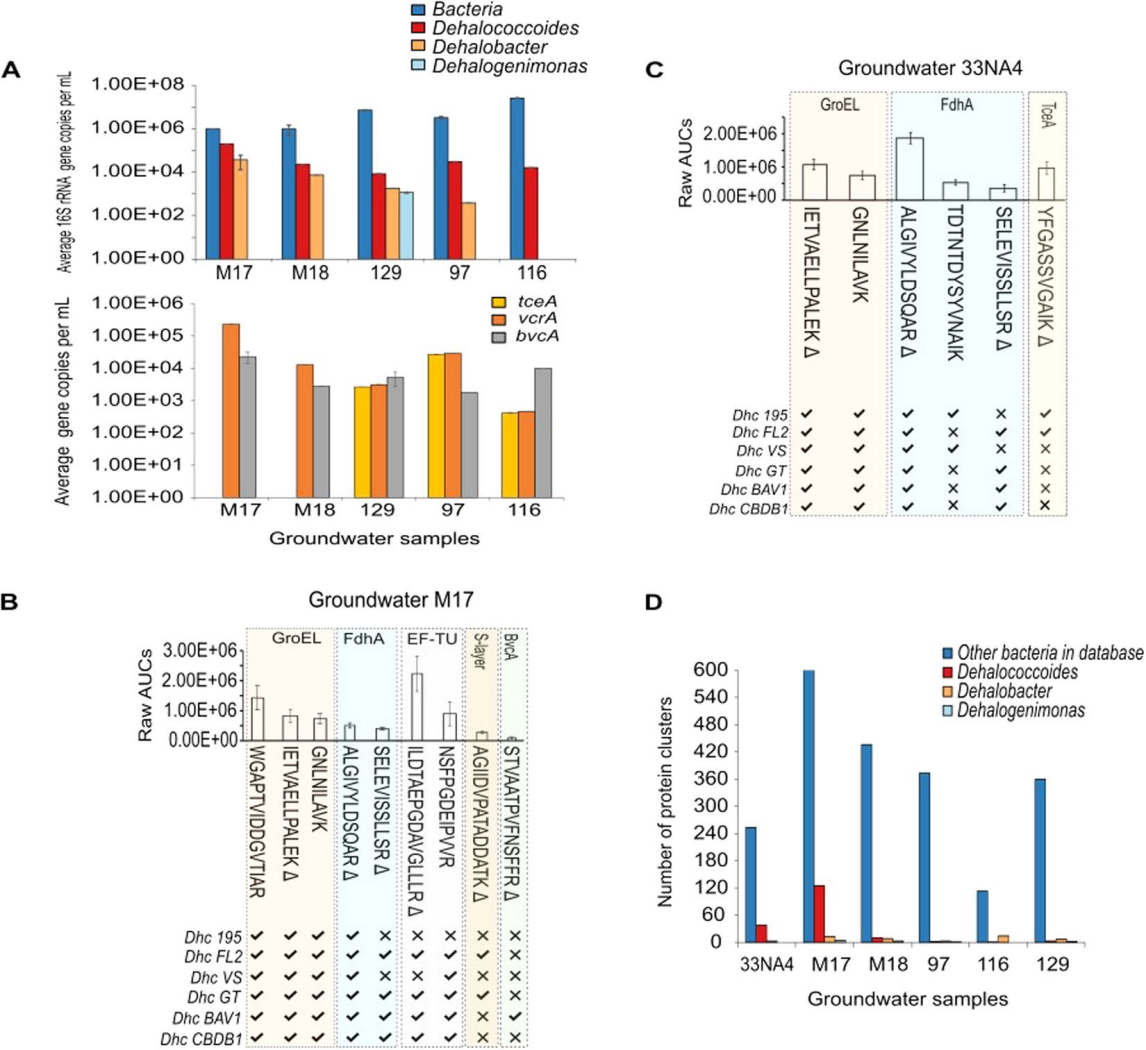
*Dhc* biomarker identification in groundwater samples. (**A**) qPCR measurements of bacterial, *Dhc*, *Dehalobacter* and *Dehalogenimonas* 16S rRNA gene copy numbers. Gene copy numbers of *tceA* and *bvcA* are also shown. Values are given on a log scale and each bar represents one DNA extraction quantified in triplicate. DNA-based analyses were not performed for sample 33NA4 due to limited availability. *tceA* genes were detected but not quantifiable in samples M17 and M18. (**B**,**C**) Average raw peak area under the curve (AUC) values of the targeted peptides identified in LC-MRM-MS runs of tryptic digests from groundwater samples M17 and 33NA4, respectively. Error bars are the standard error of the mean (*n* = 3 technical replicates). Peptides marked with Δ were identified with supporting evidence from spiked-in unlabeled standards. The inserts below each graph show the specificities of the peptides, determined *in-silico*, to the proteomes of the six most common isolates of *Dhc* bacteria. (**D**) Total number of proteins by bacterial genus analyzed by qPCR that were identified by global proteomics analyses of groundwater samples. A detailed list of proteins per sample is presented in Supplementary Table S8.

qPCR measurements performed on groundwater samples M17, M18, 97, 116, and 29 (33NA4 samples for DNA extraction were not available) showed average total bacterial 16S rRNA gene copies/mL values ranging from 2.6 × 10^7^ ± 1.4 × 10^6^ in sample 116 to 9.8 × 10^5^ copies/mL ± 4.9 × 10^5^ in sample M18 (Fig. 4A). qPCR measurements of 16S rRNA genes of relevant organohalide respirators (*Dhc*, *Dehalobacter*, and *Dehalogenimonas*) demonstrated the presence of *Dhc* bacteria in all samples, with the highest abundance of *Dhc* 16S rRNA genes quantified in sample M17. At the M17 sampling location, *Dhc* represented ~20% of the total bacterial 16S rRNA genes (2.0 × 10^5^ ± 1.7 × 10^3^ copies/mL). According to empirical information from bioremediation site regulators, they have found that > 1 × 10^5^ copies/mL of organohalide respirators such as *Dhc* are needed for observable dechlorination, to occur. Thus, the qPCR data above suggested that M17 has appropriate *Dhc* cellular abundance for dechlorination, whereas the other samples has cellular abundances that appear to be below this minimum threshold.

As discussed previously, the identification of *Dhc* genes does not necessarily indicate that *Dhc* is actively dechlorinating TCE or any other chlorinated ethene. Amongst the reasons for this observation are the lack of correlation between dechlorination activity and the abundance of *Dhc* 16S rRNA genes and the variable translation rates of RDase transcripts observed in pure and mixed cultures^12,16^. Additionally, in groundwater samples, *Dhc* microorganisms may be present but not contributing significantly to dechlorination processes due to inhibitory mechanisms (*i.e*., the presence of perfluoroalkyl acids^40^) or competition with other organohalide-respiring bacteria having more favorable chances of growth. Due to these factors, the identification of *Dhc* protein biomarkers of dechlorination, would provide more definitive information about whether Dhc active involvement in the dechlorination processes in these samples.

Analysis of the groundwater samples included in this study by targeted proteomics, identified *Dhc* biomarker proteins and peptides only in groundwater samples M17 and 33NA4 (Fig. 4B,C). A few of the other samples were viscous and consisted of black oily, sticky material that complicated filtering in Sterivex cartridges and potentially limited DNA and protein extraction and subsequent measurement. This may be an issue in general sampling at some sites but was beyond the scope of this manuscript.

GroEL proteins were observed in both M17 and 33NA4 samples and were identified by peptides that are highly conserved across the proteomes of multiple *Dhc* strains, including those of the six isolates (Fig. 4B,C). Besides detection of GroEL in both M17 and 33NA4 samples, targeted peptides from the housekeeping EF-TU and structural S-layer biomarkers were also detected in groundwater M17 (Fig. 4B). For example, the EF-TU peptide ILDTAEPGDAVGLLLR, which differs by a single threonine residue compared to the peptide identified in consortium BDI (Fig. 3), and is present in multiple *Dhc* strains, demonstrated the utility of targeted proteomics to differentiate single amino acid changes in the sequences of the analytes. The additional detection of the S-layer peptide AGIIDVPATADDATK in sample M17, which is found in four *Dhc* proteomes, including those of strains GT and FL2, also suggested that specific *Dhc* strains were present in this sample.

Evidence of dechlorination activity was obtained by the detection of two and three FdhA peptides in samples M17 and 33NA4, respectively (Fig. 4B,C). Common between both samples was the detection of the FdhA peptides ALGIVYLDSQAR and SELEVISSLLSR, which can be found in 25 and 19 *Dhc* proteomes, respectively, of the 31 *Dhc* proteomes available in UniProt (as of July 2018). Peptide ALGIVYLDSQAR has been selected as MRM target for absolute protein abundance quantification in published reports examining pure and mixed *Dhc* cultures^7,18^, which also points to its high conservation amongst *Dhc* strains and robust characteristics for mass spectrometric analyses. In addition to the ALGIVYLDSQAR and SELEVISSLLSR peptides, the detection of the FdhA peptide TDTNDYSYVNAIK in groundwater sample 33NA4 suggested that organisms representing *Dhc* strains 195, KBTCE2, CG4 and KBTCE3, were involved in active dechlorination.

Supporting the FdhA observations in samples M17 and 33NA4 and hence, the potential of active dechlorination, we also identified a TceA peptide in sample 33NA4 and a BvcA peptide in M17. For instance, the TceA peptide YFGASSVGAIK in sample 33NA4 (Fig. 4C) suggested the involvement of *Dhc* strains expressing the *tceA* RDase (e.g., strains 195 and FL2) in the dechlorination reactions leading to the transformation of TCE to VC and ethene. Similarly, the BvcA peptide STVAATPVFNSFFR in sample M17 (Fig. 4B), pointed to active transformation reactions of *cis*-DCE to ethene by strain BAV-type *Dhc*.

The data provided by LC-MRM-MS thus contrasted with the initial qPCR information, in which *Dhc* 16S rRNA genes were detected in all groundwater samples, but peptides of the targeted proteins were not identified in four of them (M18, 97, 116, or 129). This suggested that either the targeted proteins were not expressed in these samples, the proteins were of too low abundance to be detected by targeted proteomics, or the enzymatic digestion of the proteins in a sample could have produced a different set of peptides to the ones targeted. To provide insight into these issues, high-mass-accuracy and high-mass-resolution global proteomics data was also collected. For this purpose, the proteomes of six *Dhc* isolates and other bacteria that have been isolated from aquifers or sediment material contaminated with organic chlorinated compounds were combined into a database for MS spectra search (Supplementary Table S7).

Global proteomics revealed that the samples having the highest numbers of *Dhc* protein identifications were samples M17 (125 groups) and 33NA4 (38 groups), in which peptides from *Dhc* biomarkers were also detected by LC-MRM-MS (Fig. 4D). Indeed, the *Dhc* dechlorination biomarkers BvcA for sample M17, TceA for 33NA4, and FdhA for both, were also identified in the global proteomics datasets (Supplementary Table S8). The detection of TceA in sample 33NA4 and the absence of *Dhgm* proteins by global analyses suggested that the YFGASSVGAIK peptide detected before by targeted proteomics had a *Dhc* origin. We also observed that except for S-layer proteins that were identified by a different set of peptides to the ones targeted in samples 129 and 116, all the other *Dhc* biomarkers were not detected by means of global proteomics analyses in samples 129, 116 and 97 (Supplementary Table S8) which largely agreed with the targeted proteomics results. In groundwater M18, instead, *Dhc* GroEL, EF-TU and S-layer proteins were identified but with a different set of peptides. The low numbers of *Dhc* protein groups detected in samples M18, 129, 116 and 97, which included proteins that are not directly involved in mediating dechlorination processes, in combination with the aforementioned *Dhc* 16S rRNA gene data, suggested that *Dhc* cells were present but not actively dechlorinating in these samples or expressing levels of proteins that fall below the detection limits of the proteomics approach.

## Conclusions

This work demonstrates that the identification of *Dhc* biomarker proteins in contaminated groundwater through targeted proteomics is feasible. Although the approach presented here requires further optimization to provide absolute protein abundance metrics (*i.e*., molar amounts), the panel of proteins and peptides selected should be useful for further development of a robust quantitative assay. For example, three or four peptides per targeted protein (those providing the adequate MS1 characteristics, sensitivities and lower limits of detection and quantification) could be monitored in an assay, alongside isotopically labeled standards, in order to determine their absolute endogenous molar amounts.

Successful implementation of targeted proteomics for *Dhc* containing groundwater, in comparison to pure or mixed anaerobic bacterial cultures, requires knowledge of the specificity of the peptides selected from *Dhc* biomarkers in a broader microbiological context. The *in-silico* peptidome analyses conducted in this study suggested that a panel of *Dhc* specific and semi-specific peptides (albeit, found in other bacteria with dechlorination capabilities) from proteins relevant to dechlorination activities (FdhA and RDases), should be used in concert to provide a more accurate identification of *Dhc* in environmental samples.

Regarding this last point, environmental studies utilizing targeted proteomics to monitor the presence and infer the activity of *Dhc* bacteria in contaminated groundwater need to define the goal of their research – *i.e*., is it important to know the presence of active dechlorination in general, or is it also necessary to provide strain resolution? These are distinct questions – in many cases, the former question can take precedence at sites impacted with specific chlorinated pollutants. Either way, the information provided by targeted proteomics in combination with data contributed by other well-established technologies like qPCR, can offer a more complete view of key microbes and their activities contributing to contaminant detoxification. Results from gene-centric qPCR and proteomics will provide better guidance on bioremediation decisions, assist remediation project managers to efficiently manage remediation, and provide regulators a relevant line of evidence that contaminant attenuation is occurring.

## Methods

### *Dehalococcoides mccartyi* cultures, growth conditions and qPCR

Biological duplicates of actively dechlorinating axenic cultures of *Dhc* strains 195 and FL2, known to express the RDase TceA (TCE→VC and ethene), as well as strain BAV1, which expresses the BvcA RDase (DCEs→VC→ethene), were prepared and used to monitor the abundances of the targeted *Dhc* proteins in both global and targeted proteomics measurements. For the purpose of method development, the targeted proteomics approach also used a culture of the PCE-to-ethene dechlorinating Bio-Dechlor INOCULUM (BDI) consortium known to contain several *Dhc* strains and a PCE-to-*cis*-DCE-dechlorinating *Dehalobacter* strain^3^, amended with PCE as electron acceptor. Cultures were grown in completely synthetic, defined mineral salts medium as previously described^37^. Approximately 100 mL of culture (~1 × 10^10^ cells) were passed through Sterivex 0.22 µm filter units (EMD Millipore Corporation, Billerica, MA, USA) to collect the biomass. Filters were stored at −80 °C prior to protein extraction and digestion. *Dhc* cell numbers were calculated by qPCR measurements of 16S rRNA genes as previously described^6^. The known *Dhc* genomes contain a single copy of the 16S rRNA gene and RDase genes, and the gene copies measured with qPCR equal the *Dhc* cell numbers^34^.

### Groundwater samples for biomass collection

Groundwater samples from injection and monitoring wells were collected using low-flow sampling methods at three distinct sites impacted with chlorinated ethenes. Sample 33NA-4 (360 mL) was extracted from a contaminated site outside the U.S. and biomass was received on Sterivex 0.22 µm filter units. Samples M17 (745 mL) and M18 (1,350 mL) were extracted in August 2016 at a contaminated site in the United States undergoing chemical oxidation (hydrogen peroxide/chelated iron catalyst) and mineral injections, as well as supplementation of organic compounds (*i.e*., lactate) to stimulate indigenous dechlorinators. Biomass was collected *on-site* and the Sterivex 0.22 µm filter units were shipped on ice. Samples 97 (1,000 mL), 116 (962 mL), and 129 (964 mL) were collected in September 2016 from wells at a contaminated site in the United States, in which injections of emulsified vegetable oil, zero-valent iron and a *Dhc*-containing bioaugmentation consortium had occurred. The samples were collected in July 2016. Groundwater samples were received in sterile 1 L bottles and filtered through Sterivex units to concentrate the biomass immediately after arrival. All Sterivex cartridges were stored at −80 °C prior to protein extraction and digestion. Additional details regarding protein extraction and digestion procedures are provided in the Supplementary Information section.

Water quality parameters as well as chlorinated volatile organic compounds (cVOCs) and dissolved gasses concentrations were provided to us for samples M17, M18, 29, 97, and 116, as detailed in Supplementary Table S6.

### Global proteomics of axenic cultures and groundwater samples

Global proteomics measurements of the axenic cultures of *Dhc* strains 195, FL2 and BAV1 (*n* = 2 biological replicates), the BDI consortium (*n* = 3 technical replicates), as well as groundwater samples (*n* = 3 technical replicates) were obtained with an Orbitrap Q Exactive Plus mass spectrometer (Thermo Fisher Scientific, San Jose, CA) equipped with a nano-electrospray (ESI) source and interfaced with a Proxeon EASY-nLC^TM^ 1200 system. Proteolytic peptide aliquots from pure cultures (1 µg), consortium BDI (2 µg), and groundwater samples (2 µg) were suspended in solvent A (0.1% formic acid, 2% acetonitrile) and injected onto a 75 μm inner diameter microcapillary column packed with 35 cm of Kinetex C_18_ resin (1.7 μm, 100 Å, Phenomenex, Torrance, CA). Peptides were separated using a 90 minutes gradient at a flow rate of 250 nL/min from 2 to 30% solvent B (0.1% formic acid, 80% acetonitrile), followed by an increase to 40% solvent B within 10 minutes and a 10-minute equilibration with 98% solvent A. Specific details of the MS/MS data acquisition parameters have been reported previously^41^. Information concerning protein identification and analyses of datasets derived from shotgun proteomics runs are presented in the Supplementary Information section.

### Peptide selection and LC-MRM-MS method development

Initial lists of peptides (7–18 amino acids, without Methionine residues) and their transitions (+2 charged precursors, singly charged *y3* to terminal *y*-fragment series) from the targeted proteins (see Table 1) identified in data-dependent global proteomics analyses of axenic cultures of strains 195, FL2 and BAV1, were evaluated by analyzing 500 ng, 2 µg and 8 µg of total tryptic digests via LC-MRM-MS.

For each measurement, peptides were loaded onto capillary back-columns (150 µm × 120 mm) packed with ~50 mm Kinetex 5 µm C_18_ resin and chromatographically separated on in-house pulled nanospray emitters (100 µm × 170 mm) packed with ~160 mm of Kinetex 5 µm C_18_ resin. Chromatographic separation consisted of a linear gradient of solvent B (70% acetonitrile, 0.1% formic acid) at 300 nL/min from 2 to 60% within 90 minutes. After each sample run, wash/re-equilibration runs were queued. The TSQ instrument was operated with a dwell time of 20 ms, scan width set at 0.002 *m/z*, and Q1/Q3 at 0.70 full width at half maximum (FWHM). Spray voltage and capillary temperature settings in the ion source were set at 1.75 kV and 270 °C. Collision energies for each peptide were calculated using the default linear equation specific to a Thermo Scientific TSQ Ultra instrument provided in the Skyline environment.

Raw LC-MRM-MS spectral data collected were imported into the software package Skyline v3.7 (http://skyline. maccosslab.org)^42,43^ and the signals were manually analyzed to determine the quality of the peptide signals in an extracted ion chromatogram (XIC). In addition, peptide sequence specificities were also assessed *in-silico* with the Tryptic Peptide Analysis tool of Unipept 3.2 and Protein BLAST searches as described in the Supplementary Information.

### Analyses of a mixed culture and groundwater samples by LC-MRM-MS

Peptide and transitions signals selected from the microbial isolate samples were monitored in technical triplicate runs of consortium BDI and groundwater samples using the same LC-MRM-MS procedure as for the pure cultures. Amounts of tryptic digests analyzed were 4 µg for the BDI sample; 10 µg for the M17, M18, 97 and 129 groundwater samples; and 20 µg for the groundwater samples 33NA4 and 116. To validate peptide identifications in groundwater samples, we required the following criteria: (A) Co-elution of all selected transitions per peptide; (B) average dot-product (dotp) correlation scores >0.80 for transition intensity ratios between the signals detected in groundwater to those observed in the respective pure culture; and (C) peptide signal reproducibility in all technical runs.

For a subset of the target proteins (n = 6), a collective set of 11 synthetic unlabeled peptide standards were purchased as purified lyophilized solids (>95%, Thermo Scientific, Waltham, MA) and reconstituted to standard solutions in solvent A (peptides marked with Δ in Supplementary Table S3). A total of 5 pmol of each peptide standard were then spiked to 4 µg of BDI sample; 10 µg of M17, M18, 97 and 129 groundwater samples; and 20 µg of groundwater samples 33NA4 and 116. Peptide peak areas differences in samples with and without standards were used as additional validation of the presence of a peptide in a sample. In addition, we required strong agreement between the average transition ratios (dotp >0.80) and retention times (≤3 mins differences) of the endogenous peptides with the spiked-in synthetic standards. Finally, high-mass-accuracy / high-resolution global proteomics data filtered at a peptide false discovery rate (FDR) level < 1% were also used to verify the presence of all the targeted peptides and proteins in the groundwater samples.

## Supplementary information

Supplemental Information

Supplemental Table S1

Supplemental Table S2

Supplemental Table S8
