# Supplementary material for: Targeted detection of Dehalococcoides mccartyi microbial protein biomarkers as indicators of reductive dechlorination activity in contaminated groundwater
Source: Sci Rep. 2019 Jul 22;9:10604. doi: 10.1038/s41598-019-46901-6 (PMC6646388; doi:10.1038/s41598-019-46901-6)
Supplement: Supplementary file 1 — Supplemental Information [file 41598_2019_46901_MOESM1_ESM.pdf]

Supplementary Information for

Targeted detection of *Dehalococcoides mccartyi* protein biomarkers as indicators of reductive dechlorination activity in contaminated groundwater samples

Manuel I. Villalobos Solis[1,2], Paul E. Abraham[1], Karuna Chourey[1], Cynthia M. Swift[3], Frank E. Löffler[3,4,*], and Robert L. Hettich[1,*]

[1] Chemical Sciences Division, Oak Ridge National Laboratory, Oak Ridge, Tennessee 37831, United States.
[2] Department of Genome Science and Technology, University of Tennessee, Knoxville, Tennessee 37996, United States.
[3] Department of Microbiology, Department of Civil and Environmental Engineering, Department of Biosystems Engineering and Soil Science, University of Tennessee, Knoxville, Tennessee 37996, United States.
[4] Biosciences Division, Oak Ridge National Laboratory, Oak Ridge, Tennessee 37831, United States.

*Corresponding authors:

Robert Hettich
Oak Ridge National Lab, Oak Ridge, TN 37831
Email: hettichrl@ornl.gov
Phone: (865) 574-4968
Fax: (865) 241-1555

Frank E. Löffler
Department of Microbiology, University of Tennessee, Knoxville, TN 37996
Email: frank.loeffler@utk.edu
Phone: (865) 974-4933
Fax: (865) 974-4007

Table of contents

	Page
**Supplementary methods**	S3
**Sample preparation for global and targeted proteomics**	S3
**Protein identification by database searching**	S3
**Global proteomics data analysis**	S4
**RDase phylogenetic tree construction**	S4
**Unipept tools and Protein BLAST searches**	S4
**qPCR of groundwater samples**	S5
**Supplementary Figure S1**	S6
**Supplementary Figure S2**	S7
**Supplementary Figure S3**	S8
**Supplementary Figure S4**	S9
**Supplementary Figure S5**	S10
**Supplementary Table S1**	S11
**Supplementary Table S2**	S12
**Supplementary Table S3**	S13
**Supplementary Table S4**	S14
**Supplementary Table S5**	S15
**Supplementary Table S6**	S16
**Supplementary Table S7**	S17
**Supplementary Table S8**	S20
**References**	S21

Supplementary Methods

Sample preparation for global and targeted proteomics

Filtered cells from axenic cultures of *Dhc* strains 195, FL2, BAV1 (*n*=2 biological replicates), the BDI Consortium, as well as the M17, M18, 97, 116 and 129 groundwater samples (*n=1*) were processed by adding 2 mL of SDS lysis buffer (4% SDS in 100 mM Tris-HCl, pH 8.0) to the Sterivex cartridges followed by incubation in a water bath at 97ºC for 15 minutes and incubation at room temperature for 1 hour. The SDS lysis buffer was recovered and the filters rinsed once more with fresh lysis buffer. As previously described, proteins were extracted from cell lysates by trichloroacetic acid (TCA) precipitation and proteolytically digested with trypsin following denaturation and disulfide bonds being reduced and blocked.[1]

Frozen filter membranes with biomass from the 33NA4 groundwater sample (*n=1*) were removed from the cartridges and cut into ~ 1 cm pieces using a sterilized razor blade and then suspended in 5 mL of SDS lysis buffer (5% SDS in 50 mM Tris-HCl, pH 8.5; 0.15 M NaCl, 0.1 mM EDTA; 1mM $MgCl_2$; 50 mM DTT). Cells were heat-lysed as described earlier[2] and the supernatant containing the whole cell lysate transferred to new tubes. Proteins were then precipitated by TCA. Lysate mixes were centrifuged at 21000 g x 20 min to obtain a protein pellet which was washed with chilled acetone, air dried and solubilized in 6M guanidine buffer[3]. Following protein solubilization, proteolysis was initiated using trypsin. All peptide solutions were desalted on 200µL C_{18} stage tips (Thermo Scientific, Waltham, MA) and stored at -80ºC prior to global proteomics analysis. For targeted proteomics runs, volumes of processed samples were loaded directly onto capillary back columns and desalted off-line.

Protein identification by database searching

Tandem MS spectra from pure cultures of *Dhc* strains 195, FL2, BAV1, and the BDI consortium culture were searched against individual or concatenated databases of *Dhc* strains downloaded from UniProt (for strains 195, GT, VS, CBDB1, BAV1 02/2017). The IGS Annotation Engine was used for structural and functional annotation of the *Dhc* strain FL2 protein sequences (http://ae.igs.umaryland.edu/cgi/index.cgi, Reference: PMID:21677861) and the web-based tool Manatee was used to view and download protein annotations (http://manatee.sourceforge.net/). The tryptic digest of the BDI consortium was searched with a database assembled from the proteomes of the six strains of *Dhc* and *Dehalobacter restrictus* DSM 9455 (SI Table S5). Spectral data collected from groundwater samples were searched against a database encompassing the proteomes of bacterial isolates known to coexist with *Dhc* or known to inhabit aquifer and sediments (SI Table S6).

In addition to common contaminant proteins, the reversed protein sequences were appended and used as decoys to discern the false-discovery rate (FDR) at the spectral level. For standard database searching, the tandem fragmentation spectra (MS/MS) were searched with Myrimatch v2.2 algorithm[4] set to parameters described before.[5] Resulting peptide spectrum matches were then imported, filtered and organized into proteins with IDPicker v.3.1[6] software. To achieve a final peptide-level confidence > 99% (or false discovery rate FDR < 1%), proteins were identified with at least two distinct peptides sequences and a minimum spectra of 2 per protein.

Global proteomics data analysis

Protein intensity values from each global proteomics dataset were calculated by summing together the MS1-level intensities of peptide precursors that were derived from IDPicker using IDPQuantify.[7] Extracted ion chromatograms (XICs) were identified using ± 30 s lower and upper retention time tolerance and ± 10 ppm lower and upper chromatogram tolerance. Protein abundance values were normalized by dividing the protein intensity values by their length (*i.e.*, number of amino acids), performing a log2 transformation, and mean central tendency adjusted with the software platform Inferno RDN (https://omics.pnl.gov/software/infernordn).

Using the Perseus software,[8] we removed proteins in pure cultures of *Dhc* strains 195, FL2 and BAV1 that were stochastically sampled by requiring quantified proteins to be observed in both biological replicates per strain. For the BDI consortium and groundwater sample sets (*n*= 2 and 3 technical replicates, respectively) proteins observed in at least one run were considered for comparison to targeted results as their sporadic identification by global proteomics may have been due to their low biological abundances and thus we could have a probability of observing them employing LC-MRM-MS. Missing values were then imputed with random numbers from a simulated Gaussian distribution of low abundant proteins (down-shift value of 2.5 and width of 0.3). All proteins identified by LC-MS/MS were clustered at > 85% amino acid sequence identity with the UClust algorithm of the analysis tool USearch v10.0.[9] Venn diagrams were generated with the web application jvenn (http://jvenn.toulouse.inra.fr/app/index.html).

RDase phylogenetic tree construction

To provide insight into the diversity of the RDases sequences present in the proteomes of *Dhc* strains 195, FL2, and BAV1, their phylogenetic relationships were evaluated with the software MEGA 7.[10] A total of 52 RDases and two outgroup RDase sequences from *Desulfitobacterium hafniense* strain Y51 *(*Q8L172) *and Dehalobacter restrictus* DSM 9455 (AHF10441) were aligned with the MUSCLE algorithm.[11] All columns in the alignment of the protein sequences containing gaps and missing data were eliminated, leaving a total of 56 amino acid positions in the final dataset. A phylogenetic tree using the Maximum Likelihood algorithm based on the JTT matrix-based model was then constructed.[10,12] Initial trees for the heuristic search were obtained automatically by applying Neighbor-Join and BioNJ algorithms to a matrix of pairwise distance estimates using a JTT model, and then selecting the topology with superior log likelihood value. Estimation of the relative confidence scores in phylogenetic groups were determined by using 1000 bootstrap replications of the data set.[13] The tree was rooted with the outgroup RDase sequences from *Desulfitobacterium hafniense* strain Y51 and *Dehalobacter restrictus* DSM 9455.

Unipept tools and Protein BLAST searches

The Peptidome Clustering tool of the web application Unipept 3.2[14] was used to compare the percentages of pairwise similarity between the *in-silico* generated peptidomes of six *Dhc* strain proteomes databases (i.e., *Dhc* strains 195, FL2, VS, GT, BAV1, and CBDB1) against the peptidomes of representative bacterial isolates that have been obtained from groundwater, aquifer, sediment, or soil (see SI for additional information). Peptidome similarity percentages were calculated based on the minimum similarity method and then clustered by the UPGMA

algorithm. To assess if other protein records stored either at UniProt or NCBI could produce the selected peptides before monitoring in groundwater, *in-silico* specificities were evaluated using the Tryptic Peptide Analysis tool of UniPept 3.2 (equating isoleucine and leucine residues) and Protein BLAST searches against non-redundant protein sequences (replacing the N-terminus of each peptide with either K or R residues, respectively). Peptides were deemed as *Dhc*-specific if they were not found in the proteins of any other bacterial species by means of both *in silico* searches

qPCR of groundwater samples

Sterivex 0.22 µm filter units were used to concentrate biomass for groundwater samples M17, M18, 97, 116 and 129 using volumes equivalent to 225 mL, 553 mL, 1000 mL, 1000 mL and 1000 mL, respectively. DNA was isolated from the Sterivex cartridges using the standard protocol of the MoBio PowerLyzer PowerSoil Kit (MoBio, Carlsbad, CA). Quantification of DNA concentrations was conducted using the Qubit dsDNA BR Assay (Life Technologies, Grand Island, NY) as per the manufacturer's instructions. DNA solutions were stored at -80°C until qPCR measurements.

qPCR analyses targeting 16S rRNA genes from total bacteria, *Dehalococcoides* sp, *Dehalobacter* sp. And *Dehalogenimonas* sp. were conducted using a QuantStudio 12K Flex Real-Time PCR System. The primer probes for each gene targeted assay were designed using Primer Express version 3 and Geneious versions R6-R11.[15] Sample dilutions of 1:10, 1:100 and 1:1000 were prepared using nuclease-free water to determine the presence of any interfering contaminants. The *Dhc* 16S rRNA gene assay was chosen to demonstrate any contaminant interference for each sample. Upon analysis of the qPCR results, the most diluted sample demonstrating absence of contaminants and that gave the best fit within the template DNA standard curve was chosen for qPCR quantification. Gene copies numbers per mL were quantified in triplicate using the method described in[16].

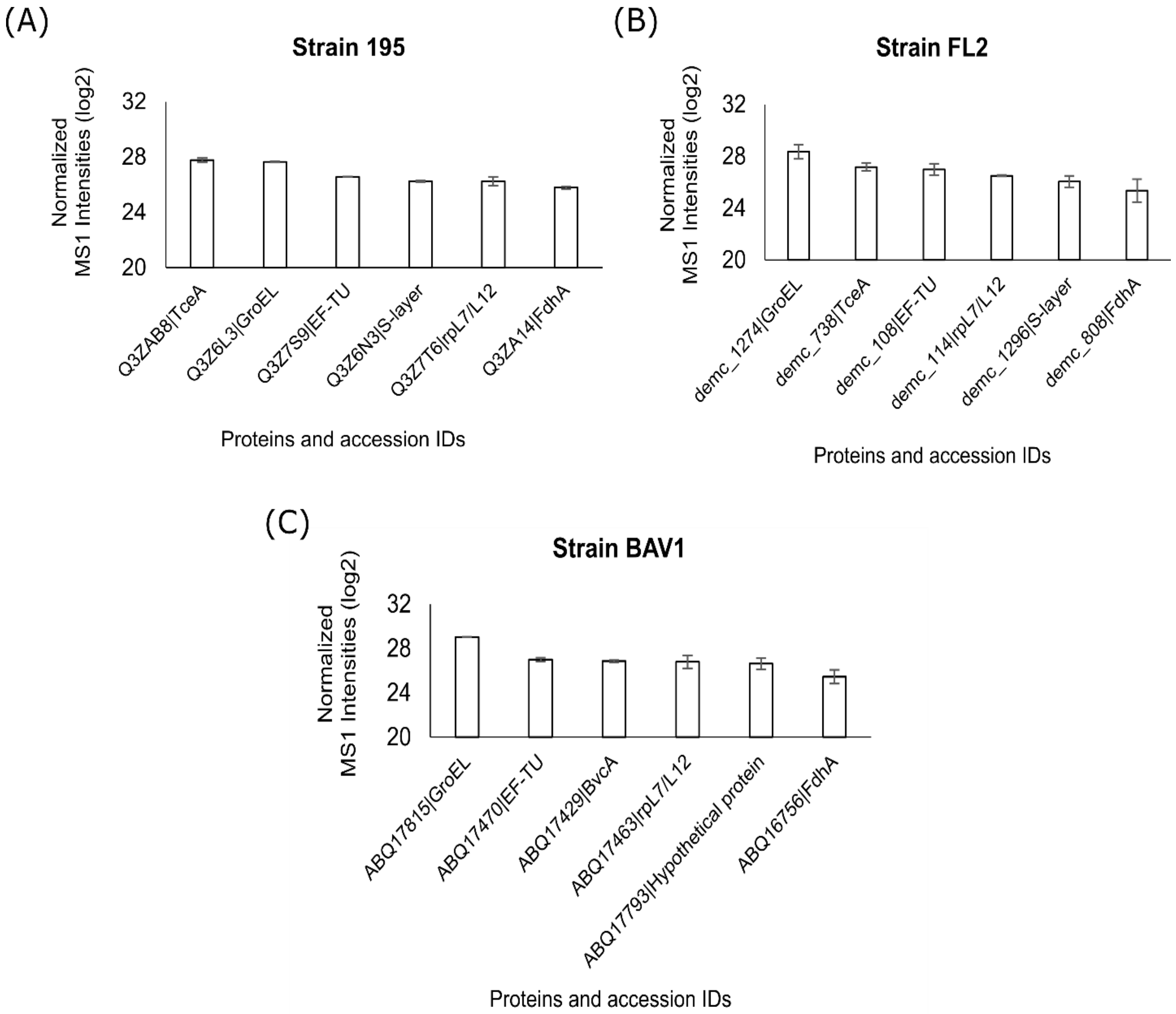

Supplementary Figure S1. Targeted protein abundances (log2 normalized MS1 intensities) in 90 mins LC-MS/MS gradients and high mass accuracy and resolution data from measurements of actively dechlorinating pure cultures of strains (**A**) 195, (**B**) FL2, and (**C**) BAV1 (*n=2* biological replicates). The accession of the S-layer protein of strain BAV1 is ABQ17793 which is annotated as a hypothetical protein in the UniProt database of strain BAV1. Error bars are the standard error of the mean.

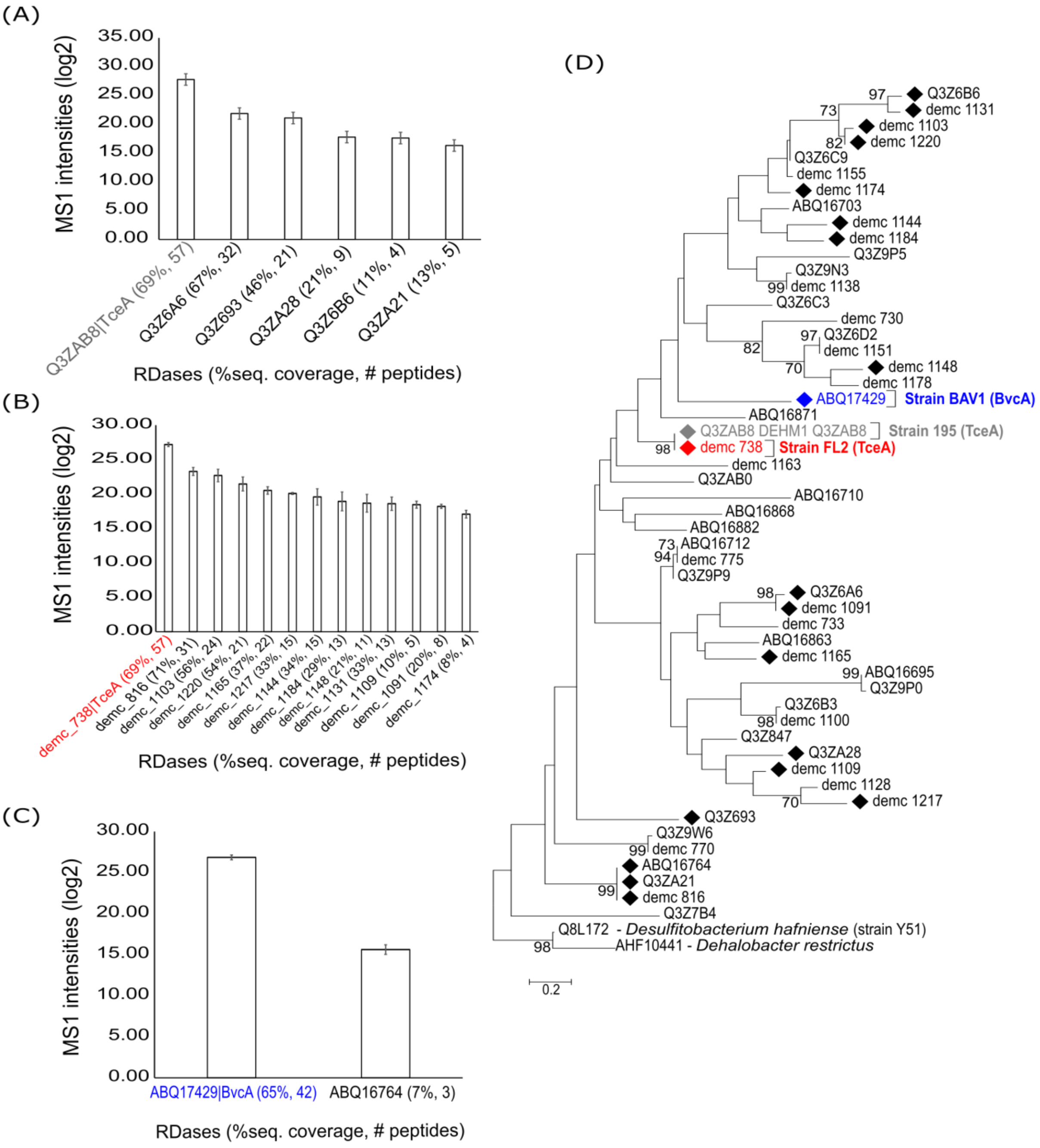

Supplementary Figure S2. Relative abundances of other identified RDases in global proteomics runs of tryptic digests of pure cultures of *Dhc* strains (**A**) 195, (**B**) FL2, and (**C**) BAV1. (**D**) Highest log likelihood (-3420.32) phylogenetic tree depicting the relationships among 52 RDases sequences in the proteomes of *Dhc* strains 195, FL2 and BAV1. Bootstrap values above 70% are shown. The tree is drawn to scale, with branch lengths measured in the number of substitutions per site. The filled diamonds represent proteins identified by global proteomic analyses. The tree was rooted with outgroup RDase sequences from *Desulfitobacterium hafniense* strain Y51 and *Dehalobacter restrictus* DSM 9455.

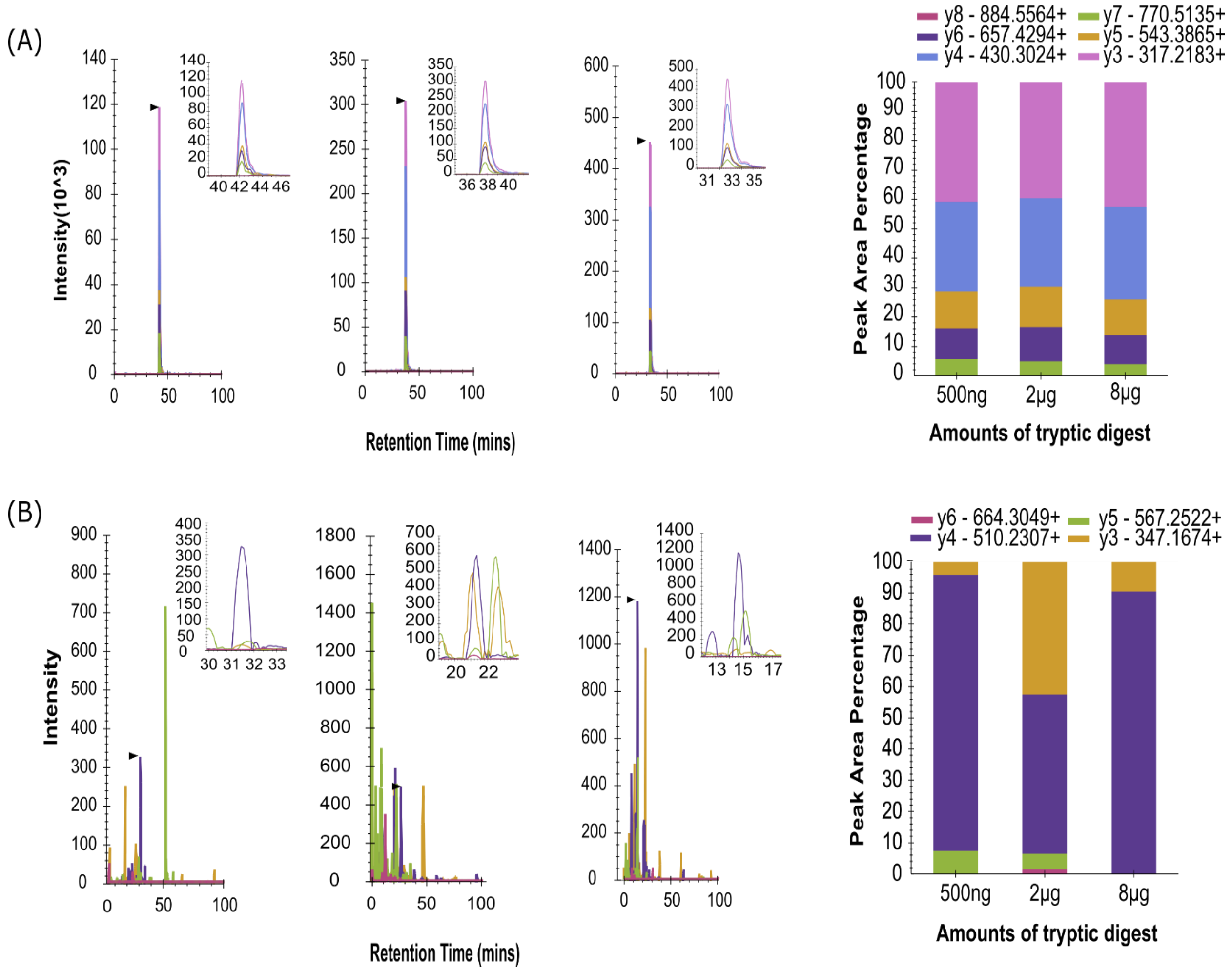

Supplementary Figure S3. XICs of the best peak groups that were automatically selected by Skyline for the GroEL peptides (**A**) GNLNILAVK (471.292 *m/z* ++) and (**B**) APGYGDR (368.174 *m/z* ++) identified in runs of 500ng, 2 µg and 8 µg (left to right) from the tryptic digest of a *Dhc* strain FL2 culture. The inserts in each XIC are the magnifications of the peaks marked by arrowheads. Each transition (precursor→ fragment ion pair) is identified by different colors. The images on the right side show the relative contribution of each fragment ion to each peptide peak marked with arrowheads. In this case, peptide APGYGDR was removed from further analysis.

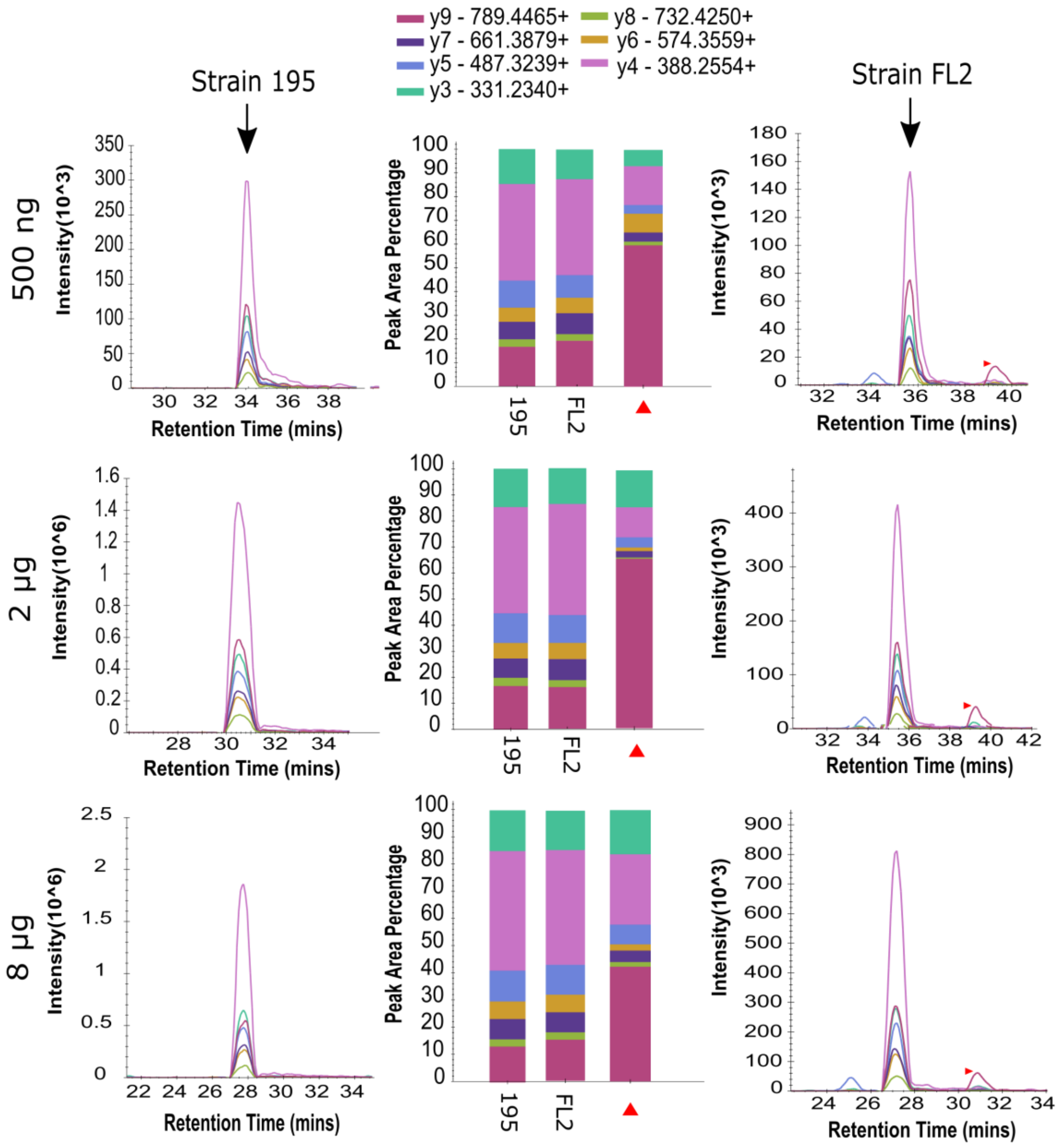

Supplementary Figure S4. Example of MRM signal identity validation between shared peptides in *Dhc* protein homologues. The chromatographic traces of peptide YFGASSVGAIK (550.292 *m/z* ++) found in expressed TceA homologues of *Dhc* strains 195 and FL2, and identified in MRM runs of 0.5 µg, 2 µg and 8 µg of their respective tryptic digests, are shown. The relative contributions of each fragment ion to the total peak areas are displayed with different colors. Other peak groups (*i.e*., the one marked with a red arrowhead) were also observed in the XICs of strain FL2 but were determined to be interfering signals when the XICs of the same peptide in strain 195 was analyzed.

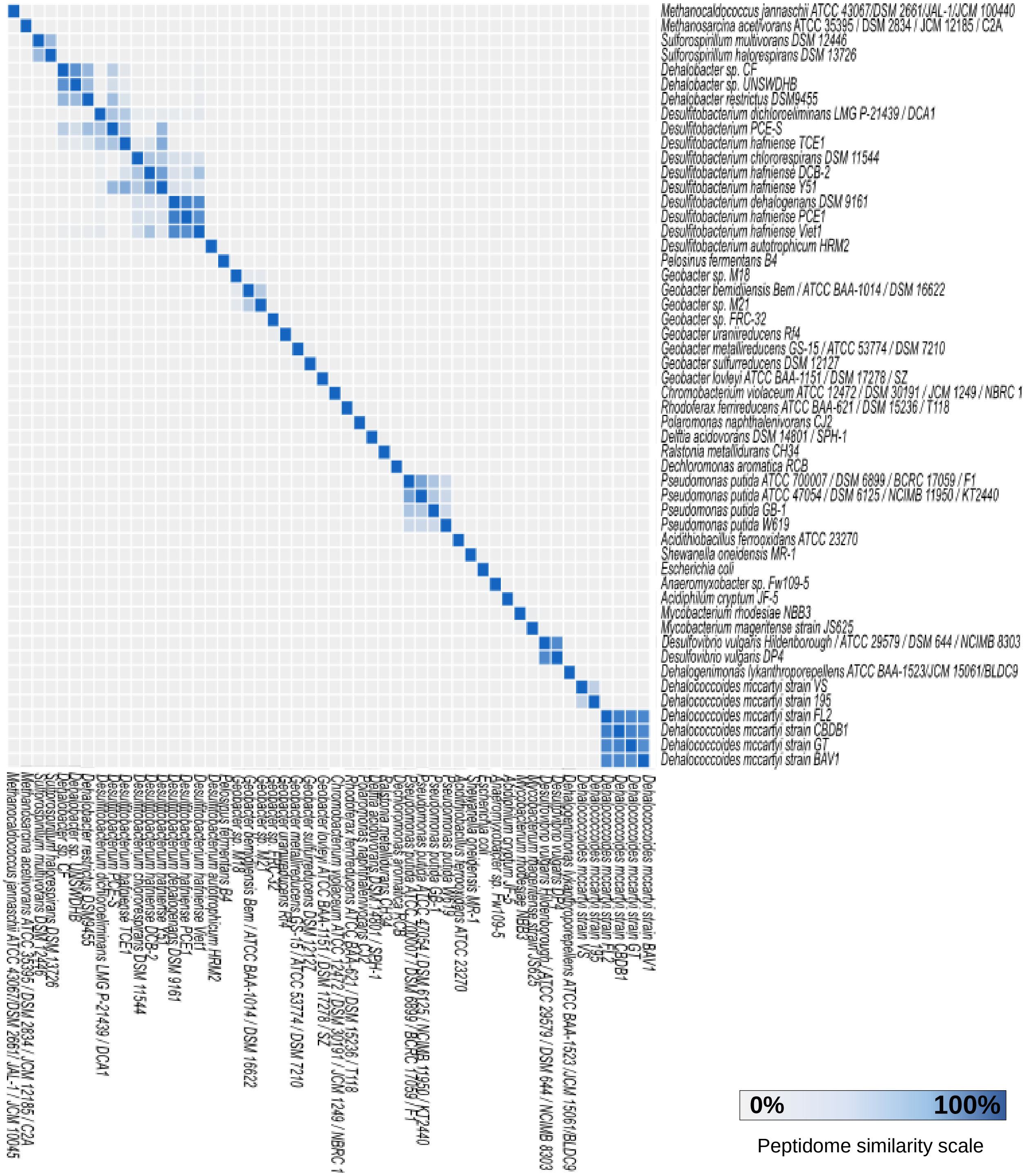

Supplementary Figure S5. Similarity matrix calculated with the Unique Peptide Finder application of Unipept 3.2 (https://unipept.ugent.be/) between the tryptic peptidomes of cultured strains of *Dhc* (including the three analyzed here) and other bacteria that have been isolated or identified in groundwater or aquifer materials. The *E. coli* proteome was also added as distant comparison

Supplementary Table S1

Provided as a separate excel file.

Supplementary Table S2

Provided as a separate excel file.

Supplementary Table S3. Top five transitions ranked by contribution to total AUC from the final set of peptides selected in pure cultures of *Dhc*.

Protein	Accessions in *Dhc* 195, FL2 and BAV1 databases	Peptide sequences	Precursors *m/z* (all doubly charged)	Fragment ions *m/z* (*y-fragment number*, all singly charged)
GroEL	Q3Z6L3, demc_1274, ABQ17815	ΔGVDTLANTVR	523.285	889.473 (y8), 774.446 (y7), 673.399 (y6), 560.315 (y5), 489.278 (y4)
		WGAPTVIDDGVTIAR	785.914	959.515 (y9), 846.431(y8), 731.404 (y7), 616.377 (y6), 460.287 (y4)
		ΔIETVAELLPALEK	713.413	983.5772(9), 783.4975(y7), 670.4134(y6), 557.3293(y5), 460.2766(y4), 389.2395(y3)
		GNLNILAVK	471.292	770.5135 (y7), 657.4294 (y6), 543.3865(y5), 430.3024(4), 317.2183(y3)
		AQIEETESAFDR	698.323	1196.5430(y10), 1083.4589(y9), 954.4163(y8), 825.3737(y7), 595.2835(y5), 508.251 (y4)
		LEGDEATGVSIVR	673.351	1103.5691(y11), 802.4781(y8), 731.4410(y7), 630.3933(y6), 474.3035(y4)
FdhA	Q3ZA14, demc_808, ABQ16756	SWDWALGEIANK	695.343	815.4621 (y8), 744.4250(y7), 631.3410+ (y6), 445.2769 (4), 332.1928 (y3)
		Δ ALGIVYLDSQAR	653.361	951.4894(y8), 852.4210(y7), 689.3577(y6), 576.2736(y5), 461.2467(y4)
		Δ †GTELISVDCR	575.282	862.4451(y7), 749.3610(y6), 636.2770(y5), 549.2450(y4), 450.1765(y3)
		SGSEIAFTGGLIK	640.348	806.4771(y8), 735.4400(y7), 588.3715(y6), 487.3239(y5), 430.3024(y4), 373.2809+(y3)
		SSEQNAASLLK	574.301	716.4301(y7), 602.3872(y6), 531.3501(y5), 460.3130(y4), 373.2809(y3)
		TDTNTDYSYVNAIK	802.875	794.4407(y7), 707.4087(y6), 544.3453(y5), 445.2769(y4), 331.2340(y3)
		†SELEVISSLFSR	683.864	1037.5626(y9), 908.5200(y8), 809.4516(y7), 696.3675(y6), 609.3355(y5)
		GSAGEYPVICTTVR	755.371	1108.5819(y9), 945.5186(y8), 749.3974(y6), 636.3134(y5), 476.2827(y4)
		LSTASSLEALAASFGR	790.917	921.4789(y9), 792.4363(y8), 608.3151(y6), 537.2780(y5), 466.2409(y4)
		SGSEIAFIGGLIK	646.366	818.5135(y8), 747.4763(y7), 600.4079(y6), 487.3239(y5), 430.3024(y4), 373.2809(y3)
		†TDNNTNYSYINAIK	815.889	808.4563(y7), 721.4243(y6), 558.3610(y5), 445.2769(y4), 331.2340(y3)
		SELEVISSLLSR	66.872	1003.5782(y9), 874.5356(y8), 775.4672(y7), 662.3832(y6), 575.3511(y5)
		VCAFFAATGK	536.268	741.3930(y7), 594.3246(y6), 447.2562(y5), 376.2191(y4), 305.1819(y3)
TceA	Q3ZAB8, demc_738	ϕDVDDLLSAGK	516.764	818.4254+ (y8), 588.371 (y6), 362.203 (y4), 275.171 (y3)
		ϕLEIELQGK	465.268	816.4462(y7), 687.4036(y6), 574.3195(y5), 445.2769(y4), 332.1928(y3)
		Δ ϕYFGASSVGAIK	550.292	789.4465(y9), 661.3879(y7), 487.3239(y5), 388.2554(y4), 331.2340(y3)
BvcA	ABQ17429	ϕDLYLAWAK	490.266	751.4137(y6), 588.3504(y5), 475.2663(y4), 404.2292(y3)
		Δ ϕTPVPIVWEEVDK	706.377	904.4411(y7), 805.3727(y6), 619.2933(y5), 490.2508(y4), 361.2082(y3)
		Δ ϕSTVAATPVFNSFFR	772.398	1185.6051(y10),1013.5203(y8), 817.3991(y6), 670.3307(y5), 556.2878(y4)
EF-TU	Q3Z7S9, demc_108, ABQ17463	NSFPGDEIPIVR	672.351	995.5520(y9), 898.4993(y8),726.4509(y6), 597.4083(y5), 484.3242(y4)
		ϕILDSAEPGDAVGLLLR	819.9567	1010.5993(y10), 741.4981(y7), 670.4610(y6), 571.3926(y5), 401.2871(y3)
		NSFPGDEIPVVR	665.3435	981.5364(y9), 884.4836(y8), 712.4352(y6), 583.3926(y5), 470.3085(y4)
		Δ ILDTAEPGDAVGLLLR	826.9645	1010.5993(y10), 741.4981(y7), 670.4610(y6), 571.3926(y5), 401.2871(y3)
S-layer	Q3Z6N3, demc_1296, ABQ17793**	†YFGNQWNQTALCK	815.377	834.4138(y7), 720.3709(y6), 592.3123(y5), 491.2646(y4), 420.2275(y3)
		†AGIIPAPTTASDAYK	738.39	953.4575(y9),755.3570(y7), 654.3093(y6), 583.2722(y5), 381.2132(y3)
		†VAYGTTTGTETTATTLK	858.438	735.4247(y7), 634.3770(y6), 533.3293(y5), 462.2922(y4), 361.2445(y3)
		Δ FYDVGILEWNADK	785.382	875.4258(y7), 762.3417(y6), 633.2991(y5), 447.2198(y4), 333.1769(y3)
		TAVYATAVYDDGDDTLVR	972.962	1168.511(y10),1005.448(y9),603.346(y5),488.319(y4)
		YFGNQWNQPATCK	815.377	1004.461(y8),818.382(y7),576.281(y5),479.228(y4),408.191(y3)
		TWYSADGLTFTK	695.337	1102.5415(y10), 939.4782(y9), 666.3821(y6), 496.2766(y4), 395.2289(y3)
		Δ AGIIDVPATADDATK	729.377	889.4262(y9), 721.3363(y7), 549.2515(y5), 434.2245(y4), 319.1976(y3)
		VCYGLPTGTDTIEATTLK	970.487	1347.7002(y13), 662.3719(y6), 533.3293(y5), 462.2922(y4), 361.2445(y3)

† Peptide found in one or two *Dhc* proteomes.

ϕPeptide found in protein homologues from other bacterial species (*i.e*., *Dehalogenimonas lykanthroporepellens* BL-DC-9*)*

Δ Unlabeled peptide standards available (>95% purity).

Supplementary Table S4. Average dotp correlation values (*n*= 3 technical replicates) obtained for all peptides identified in BDI and groundwater samples M17 and 33NA4.

Sample	Protein	Peptide	Average Raw Peak Areas (u)	Average Retention Times (mins)	Average dotp to strain 195[a]	Average dotp to strain FL2[a]	Average dotp to strain BAV1[a]	Average dotp to spiked samples[b]
	GroEL	GVDTLANTVR	3.68E+06	24.96	0.97	0.97	0.96	0.95
		WGAPTVIDDGVTIAR	3.56E+06	47.61	0.97	0.96	0.97	*NA*
		IETVAELLPALEK	1.63E+06	61.96	0.94	0.94	0.95	0.95
		GNLNILAVK	1.77E+06	37.05	0.92	0.92	0.93	*NA*
		AQIEETESAFDR	1.15E+06	28.24	0.82	0.77	0.82	*NA*
		LEGDEATGVSIVR	4.28E+05	29.52	0.88	*NP*	*NP*	*NA*
**BDI**	*FdhA*	TDTNTDYSYVNAIK	2.13E+05	36.10	0.82	*NP*	*NP*	*NA*
		GSAGEYPVICTTVR	6.72E+05	33.77	0.91	*NP*	*NP*	*NA*
		ALGIVYLDSQAR	1.77E+06	43.78	0.95	0.95	0.95	0.95
		SELEVISSLLSR	1.95E+06	66.59	*NP*	0.92	0.92	0.94
	TceA	YFGASSVGAIK	5.57E+05	32.64	0.89	0.89	*NP*	0.90
	EF-Tu	NSFPGDEIPIVR	2.09E+06	44.91	0.89	*NP*	*NP*	*NA*
		ILDSAEPGDAVGLLLR	4.14E+05	59.55	0.96	*NP*	*NP*	*NA*
	GroEL	GNLNILAVK	7.42E+05	31.98	0.93	0.94	0.94	*NA*
		IETVAELLPALEK	8.28E+05	60.08	0.91	0.91	0.90	0.90
		WGAPTVIDDGVTIAR	1.43E+06	44.70	0.96	0.97	0.98	*NA*
**M17**	*FdhA*	ALGIVYLDSQAR	5.07E+05	39.89	0.93	0.92	0.92	0.93
		SELEVISSLLSR	4.00E+05	65.32	*NP*	0.92	0.92	0.92
	BvcA	STVAATPVFNSFFR	9.24E+04	55.76	*NP*	*NP*	0.83	0.82
	EF-Tu	ILDTAEPGDAVGLLLR	2.23E+06	58.26	*NP*	*NP*	0.96	0.97
		NSFPGDEIPVVR	9.00E+05	37.98	*NP*	0.91	0.91	*NA*
	S-layer	AGIIDVPATADDATK	2.84E+05	30.23	*NP*	0.85	*NP*	0.86
	GroEL	IETVAELLPALEK	1.07E+06	66.74	0.95	0.96	0.95	0.94
		GNLNILAVK	7.48E+05	35.47	0.93	0.93	0.94	*NA*
**33NA4**	*FdhA*	ALGIVYLDSQAR	1.86E+06	43.83	0.96	0.96	0.97	0.97
		TDTNTDYSYVNAIK	5.32E+05	32.30	0.93	*NP*	*NP*	*NA*
		SELEVISSLLSR	3.61E+05	71.25	*NP*	0.91	0.92	0.92
	TceA	YFGASSVGAIK	9.65E+05	30.58	0.94	0.94	*NP*	0.94

NP, not present in sequence. *NA*, peptide standard not available.

[a] Compared against the ratios of each peptide identified in the 2 µg runs of digested pure cultures.

[b] Compared against the ratios of 5 pmol of spiked internal standard in the same sample.

Supplementary Table S5. Proteomes included in the database used to search the global proteomics data collected from consortium BDI.

Microorganisms	Proteome source	Proteome ID
Dehalobacter sp. UNSWDHB	UniProt	UP000015814
Dehalococcoides mccartyi CBDB1	UniProt	UP000000433
Dehalococcoides mccartyi (strain ATCC BAA-2266/KCTC 15142/195) (*Dehalococcoides ethenogenes* (strain 195))	UniProt	UP000008289
Dehalococcoides mccartyi FL2	Manatee	Not publicly available
Dehalococcoides mccartyi GT	UniProt	UP000002635
Dehalococcoides mccartyi VS	UniProt	UP000002506
Dehalococcoides mccartyi BAV1	UniProt	UP000002607

Supplementary Table S6. Concentrations of chlorinated volatile organic compounds (cVOCs) and dissolved gases in the groundwater samples analyzed by qPCR and proteomics. Measurements for sample 33NA4 were not available.

	Groundwater samples (date of extraction)				
Analyte (concentration units)	**M17 (08/24/16)**	**M18 (08/24/16)**	**129 (09/17/16)**	**116 (09/18/16)**	**97 (09/17/16)**
Trichloroethene (µg/L)	1	5.6	< 1.3	< 100	0.44*
cis-1,2-Dichloroethene (µg/L)	43.7	229	45.1	2440	81.2
Vinyl Chloride (µg/L)	51.7	15.5	139	47700	60
Ethene (µg/L)	140	1.7	26.4	2230	111

* The analyte was detected but the quantity result was estimated due to analyte-specific quality-control (QC) criteria discrepancies and/or the analyte was detected at a concentration between the limit of detection and quantification.

Note:

Maximum Contaminant Levels (MCLs) for Trichloroethene, *cis*-1,2-Dichloroethene and Vynil Chloride are 5 µg/L, 70 µg/L and 2 µg/L, respectively.

Supplementary Table S7. Proteomes and protein sequences included in the database used to search the global proteomics data collected from groundwater samples.

Proteomes	Proteome source	Proteome ID
Acidiphilium cryptum JF-5	UniProt	UP000000245
Acidithiobacillus ferrooxidans ATCC 23270	UniProt	UP000001362
Anaeromyxobacter sp. Fw109-5	UniProt	UP000006382
Chromobacterium violaceum ATCC 12472	UniProt	UP000001424
Delftia acidovorans SPH-1	UniProt	UP000000784
Dechloromonas aromatica RCB	UniProt	UP000000550
Desulfobacterium autotrophicum HRM2	UniProt	UP000000442
Desulfitobacterium hafniense DCB-2	UniProt	UP000007726
Desulfitobacterium hafniense Y51	UniProt	UP000001946
Desulfovibrio vulgaris vulgaris DP4	UniProt	UP000009173
Desulfovibrio vulgaris vulgaris Hildenborough	UniProt	UP000002194
Geobacter daltonii (strain DSM 22248/JCM 15807/FRC-32)	UniProt	UP000007721
Methanosarcina acetivorans C2A	UniProt	UP000002487
Methanocaldococcus jannaschii DSM 2661	UniProt	UP000000805
Pelosinus fermentans B4	UniProt	UP000004324
Polaromonas naphthalenivorans CJ2	UniProt	UP000000644
Pseudomonas putida F1	UniProt	UP000006553
Pseudomonas putida GB-1	UniProt	UP000002157
Pseudomonas putida KT2440	UniProt	UP000000556
Pseudomonas putida W619	UniProt	UP000000720
Rhodoferax ferrireducens T118	UniProt	UP000008332
Ralstonia metallidurans (strain ATCC 43123 / DSM 2839 / NBRC 102507 / CH34)	UniProt	UP000002429
Shewanella oneidensis MR-1	UniProt	UP000008186
Geobacter bemidjiensis Bem (T)	UniProt	UP000008825
Mycobacterium rhodesiae NBB3	UniProt	UP000005442
Mycobacterium mageritense DSM 44476	UniProt	UP000028867
Geobacter sp. (strain M18)	UniProt	UP000001442
Geobacter lovleyi SZ	UniProt	UP000002420
Dehalogenimonas lykanthroporepellens BL-DC-9	UniProt	UP000002349
Dehalobacter sp. UNSWDHB	UniProt	UP000015814

Dehalobacter restrictus DSM 9455	UniProt	UP000018934
Geobacter metallireducens GS-15	UniProt	UP000007073
Geobacter M21	UniProt	UP000002380
Geobacter sulfurreducens strain ATCC 51573/DSM 12127/PCA	UniProt	UP000000577
Geobacter uraniireducens (strain Rf4)	UniProt	UP000006695
Dehalococcoides mccartyi CBDB1	UniProt	UP000000433
Dehalococcoides mccartyi (strain ATCC BAA-2266/KCTC 15142/195) (*Dehalococcoides ethenogenes* (strain 195))	UniProt	UP000008289
Dehalococcoides mccartyi FL2	Manatee	
Dehalococcoides mccartyi GT	UniProt	UP000002635
Dehalococcoides mccartyi VS	UniProt	UP000002506
Dehalococcoides mccartyi BAV1	UniProt	UP000002607
Sulforospirillum multivorans DSM 12446	UniProt	UP000019322
Sulforospirillum halorespirans DSM 13726	UniProt	UP000094609
Desulfitobacterium chlororespirans DSM 11544	UniProt	UP000184010
Desulfitobacterium dehalogenans DSM 9161	UniProt	UP000006053
Desulfitobacterium dichloroeliminans strains DCA1	UniProt	UP000010797
Desulfitobacterium hafniense sp. PCE1	RefSeq	NZ_KB913023.1

**Proteins**	**Protein sequence source**	**Accession**
recombinase A, partial [Desulfitobacterium sp. Viet-1]	NCBI Protein	AAN77115.1
o-chlorophenol reductive dehalogenase [Desulfitobacterium sp. Viet-1]	NCBI Protein	AAG49544.1
D-alanine-D-lactate ligase, partial [Desulfitobacterium hafniense TCE1]	NCBI Protein	AEP96394.1
putative reductive dehalogenase RdfA, partial [Desulfitobacterium hafniense TCE1]	NCBI Protein	AAG46189.1
trigger factor 2 [Desulfitobacterium hafniense TCE1]	NCBI Protein	ADI24428.1
trigger factor 1 [Desulfitobacterium hafniense TCE1]	NCBI Protein	ADI24427.1
hypothetical protein, partial [Desulfitobacterium hafniense TCE1]	NCBI Protein	CAG70356.1
putative transposase [Desulfitobacterium hafniense TCE1]	NCBI Protein	CAG70355.1
TatA protein [Desulfitobacterium hafniense TCE1]	NCBI Protein	CAG70354.1
putative transposase [Desulfitobacterium hafniense TCE1]	NCBI Protein	CAG70353.1
putative trigger factor protein [Desulfitobacterium hafniense TCE1]	NCBI Protein	CAG70352.1
putative membrane bound regulatory protein [Desulfitobacterium hafniense	NCBI Protein	CAG70351.1

TCE1]		
putative tetrachloroethene reductive dehalogenase anchor protein [Desulfitobacterium hafniense TCE1]	NCBI Protein	CAD28793.2
putative transposase [Desulfitobacterium hafniense TCE1]	NCBI Protein	CAG70350.1
tetrachloroethene reductive dehalogenase [Desulfitobacterium hafniense TCE1]	NCBI Protein	CAD28792.1
putative protein, partial [Desulfitobacterium sp. PCE-S]	NCBI Protein	AAO60103.1
tetrachloroethene reductive dehalogenase membrane-bound subunit [Desulfitobacterium sp. PCE-S]	NCBI Protein	AAO60102.1
tetrachloroethene reductive dehalogenase precursor [Desulfitobacterium sp. PCE-S]	NCBI Protein	AAO60101.1
hypothetical protein [Desulfitobacterium sp. PCE-S]	NCBI Protein	AAO60100.1
putative transposase, partial [Desulfitobacterium sp. PCE-S]	NCBI Protein	AAO60099.1

Supplementary Table S8.

Provided as a separate excel file.

References

1 Yang, S. *et al.* Clostridium thermocellum ATCC27405 transcriptomic, metabolomic and proteomic profiles after ethanol stress. *BMC genomics* **13**, 1, doi: 10.1186/1471-2164-13-336 (2012).

2 Chourey, K. *et al.* Direct cellular lysis/protein extraction protocol for soil metaproteomics. *Journal of proteome research* **9**, 6615-6622, doi:10.1021/pr100787q (2010).

3 Giannone, R. J. *et al.* Proteomic characterization of cellular and molecular processes that enable the Nanoarchaeum equitans-Ignicoccus hospitalis relationship. *PLoS One* **6**, e22942, doi:10.1371/journal.pone.0022942 (2011).

4 Tabb, D. L., Fernando, C. G. & Chambers, M. C. MyriMatch: highly accurate tandem mass spectral peptide identification by multivariate hypergeometric analysis. *Journal of proteome research* **6**, 654-661, doi:10.1021/pr0604054 (2007).

5 Johnson, C. W. *et al.* Eliminating a global regulator of carbon catabolite repression enhances the conversion of aromatic lignin monomers to muconate in Pseudomonas putida KT2440. *Metabolic Engineering Communications,* **5**, 19-25, doi:10.1016/j.meteno.2017.05.002 (2017).

6 Ma, Z. Q. *et al.* IDPicker 2.0: Improved protein assembly with high discrimination peptide identification filtering. *Journal of proteome research* **8**, 3872-3881, doi:10.1021/pr900360j (2009).

7 Chen, Y. Y. *et al.* IDPQuantify: combining precursor intensity with spectral counts for protein and peptide quantification. *Journal of proteome research* **12**, 4111-4121, doi:10.1021/pr400438q (2013).

8 Tyanova, S. *et al.* The Perseus computational platform for comprehensive analysis of (prote)omics data. *Nature methods* **13**, 731-740, doi:10.1038/nmeth.3901 (2016).

9 Edgar, R. C. Search and clustering orders of magnitude faster than BLAST. *Bioinformatics* **26**, 2460-2461, doi:10.1093/bioinformatics/btq461 (2010).

10 Kumar, S., Stecher, G. & Tamura, K. MEGA7: Molecular Evolutionary Genetics Analysis Version 7.0 for Bigger Datasets. *Mol. Biol. Evol.* **33**, 1870-1874, doi:10.1093/molbev/msw054 (2016).

11 Edgar, R. C. MUSCLE: a multiple sequence alignment method with reduced time and space complexity. *BMC bioinformatics* **5**, 113, doi:10.1186/1471-2105-5-113 (2004).

12 Jones, D. T., Taylor, W. R. & Thornton, J. M. The rapid generation of mutation data matrices from protein sequences. *Comput. Appl. Biosci.* **8**, 275-282 (1992).

13 Felsenstein, J. Confidence Limits on Phylogenies: An Approach Using the Bootstrap. *Evolution; international journal of organic evolution* **39**, 783-791, doi:10.1111/j.1558-5646.1985.tb00420.x (1985).

14 Mesuere, B., Jeugt, F., Devreese, B., Vandamme, P. & Dawyndt, P. The unique peptidome: Taxon-specific tryptic peptides as biomarkers for targeted metaproteomics. *Proteomics* **16**, 2313-2318, doi: 10.1002/pmic.201600023 (2016).

15 Kearse, M. *et al.* Geneious Basic: an integrated and extendable desktop software platform for the organization and analysis of sequence data. *Bioinformatics* **28**, 1647-1649, doi:10.1093/bioinformatics/bts199 (2012).

16 Ritalahti, K. M. *et al.* Quantitative PCR targeting 16S rRNA and reductive dehalogenase genes simultaneously monitors multiple Dehalococcoides strains. *Appl. Environ. Microbiol.* **72**, 2765-2774, doi:10.1128/AEM.72.4.2765-2774.2006 (2006).
